# Unbalanced social–ecological acceleration led to state formation failure in early medieval Poland

**DOI:** 10.1073/pnas.2409056122

**Published:** 2025-04-21

**Authors:** Adam Izdebski, Sambor Czerwiński, Marek Jankowiak, Marcin Danielewski, Sabina Fiołna, Raphael Gromig, Piotr Guzowski, Negar Haghipour, Irka Hajdas, Piotr Kołaczek, Mariusz Lamentowicz, Katarzyna Marcisz, Jakub Niebieszczański, Paweł Sankiewicz, Bernd Wagner

**Affiliations:** ^a^Palaeo-Science and History Group, Max Planck Institute of Geoanthropology, Jena 07743, Germany; ^b^Faculty of Liberal Arts, University of Warsaw, Warsaw 00-927, Poland; ^c^Centre for Systemic Risk Analysis, University of Warsaw, Warsaw 00-927, Poland; ^d^Department of Geomorphology and Quarternary Geology, Faculty of Oceanography and Geography, University of Gdańsk, Gdańsk 81-378, Poland; ^e^Faculty of Archaeology, Adam Mickiewicz University in Poznań, Poznań 61-614, Poland; ^f^Department of Philosophy and Humanities, Institute of Greek and Latin Languages and Literatures, Freie Universität Berlin, Berlin 14195, Germany; ^g^Department of Earth Sciences, Simon Fraser University, Burnaby V5A 1S6, BC, Canada; ^h^Institute of Geology and Mineralogy, University of Cologne, Cologne 50674, Germany; ^i^Faculty of History, University of Bialystok, Białystok 15-403, Poland; ^j^Laboratory of Ion Beam Physics, ETH Zurich, Zurich 8093, Switzerland; ^k^Earth Sciences Department, ETH Zurich, Zurich 8093, Switzerland; ^l^Climate Change Ecology Research Unit, Adam Mickiewicz University, Poznań 61-680, Poland; ^m^Museum of the First Piasts at Lednica, Lednogóra 62-261, Poland

**Keywords:** social–ecological systems, environmental history, political ecology, medieval archaeology, paleoecology

## Abstract

Rapid social–ecological intensification is a recurrent feature of human history. It occurred in different forms and contexts; its outcomes may have been sustainable or transient. Until recently, such intensifications usually accompanied state formation: Consolidation of political power was often coupled with exponential increase in human exploitation of the environment of a given area. Here, we study one such case, uniquely well-documented through our rich paleoecological, archaeological, numismatic, and literary data. Triggered by the Eurasian slave trade, the first “Polish” polity was founded in Central Europe c. 900 common era. It undertook unprecedented ecological intensification in its core territory, connected with large construction projects, and engaged in rapid territorial expansion. We provide new crucial evidence on this process by publishing here a high-resolution pollen profile from a location close to the polity’s capital and by an application of social network analysis to numismatic data. This state collapsed within a few generations after its foundation. The collapse of the political elites, however, did not produce a complete social and ecological disintegration of the polity’s former core region. We thus show how collapse and continuity can remain closely intertwined. Last but not least, the rich evidence on the mechanism of the collapse reveals that successful maintenance of social–ecological intensification requires reliance on a number of cultural, economic, religious, and social networks underlying the political expansion. The polity’s elite lacked access to or failed to mobilize such networks, which led to its demise.

## Significance

To understand the planet’s current crisis, we need in-depth understanding of how social–ecological change accelerated in the past and how it failed or stabilized. Our study contributes to this field by offering detailed evidence of the ecological, economic, social, and political change in a key region of medieval Central Europe, which underwent unprecedented intensification in all of these domains in the 10th c. CE. We show how social and ecological processes accompanied the consolidation of a new political formation, in turn triggered by earlier economic developments. Further, we analyze the new state’s collapse. The success (stabilization) of the acceleration necessitated simultaneous creation or integration of several networks (political, cultural, economic, etc.), which the polity’s elite failed to achieve.

Throughout the Anthropocene controversy, it has become clear that the key to understanding the current planetary crisis is the process of acceleration of human environmental impact, which occurred on a global scale in the 20th c. common era (CE), but which can also be identified, on smaller spatial scales, throughout the Holocene (1, 2). Here, we investigate an exceptionally well-documented instance of one particular type of social–ecological acceleration: state formation (3). The emergence and consolidation of hierarchical political structures is believed to be systematically associated with increasing levels of collective action and resource extraction, profoundly transforming communities and ecosystems undergoing this dynamic process (4). However, it remains unclear what conditions are necessary for this process to produce sustainable outcomes nor how ecological changes are connected to achieving higher levels of social complexity.

**Fig. 1.**
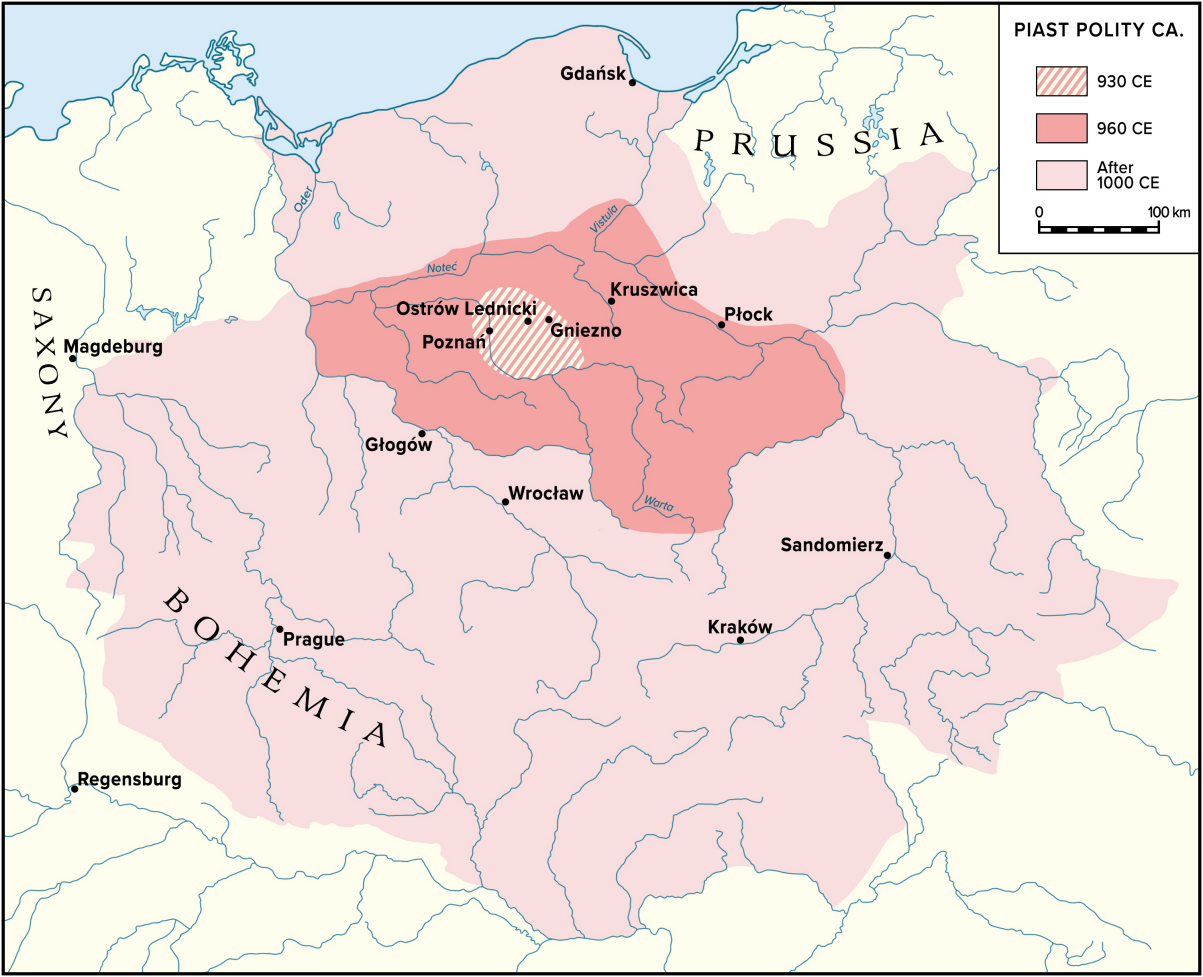
Territorial development of the first Piast polity. Based on ref. 8, p. 254, and ref. 9, p. 65.

Our case study, the emergence of the first Polish polity in Central Europe in the 10th c. CE, ruled by the Piast dynasty (Fig. 1), provides in-depth detail on all facets of this process. We combine a holistic dataset of high-resolution paleoecological, textual, numismatic, and archaeological evidence. The unique case of early medieval Central Europe, characterized by communities with anarchic lifestyles and low complexity [resulting from isolation from larger exchange networks since the fall of the Western Roman Empire in the 5th c. CE (5)], provides an exceptional backdrop for studying acceleration processes related to state formation. In the 9th and 10th c. CE, in a very short period of time and against earlier slow, linear growth trajectories, some of these communities were entering the path of rapid political expansion and ecological-economic intensification, providing modern scholars with well-defined and spatially constrained cases for studying acceleration phenomena. We focus here on one such case, which is further interesting because it was one of a few such attempts in European history that actually failed. The Piast dynasty’s first state collapsed within a few generations after its foundation (900s to 1030s CE), which led to the disintegration of the new social–ecological system it created. This meteoric rise and fall of the early Polish state presented us with the opportunity to investigate the factors that govern not only the emergence but also the longer-term sustainability of state-run social–ecological intensification. In other words, in the terminology of complex system science, which provides the theoretical underpinnings of our study (6, 7), we investigate what we call an unaccomplished, or disrupted, critical transition in regional social–ecological systems that parts of North-Central Europe experienced in the decades around the year 1000 CE.

## Rapid Intensification of Land Use Followed by a Sudden Rewilding.

**Fig. 2.**
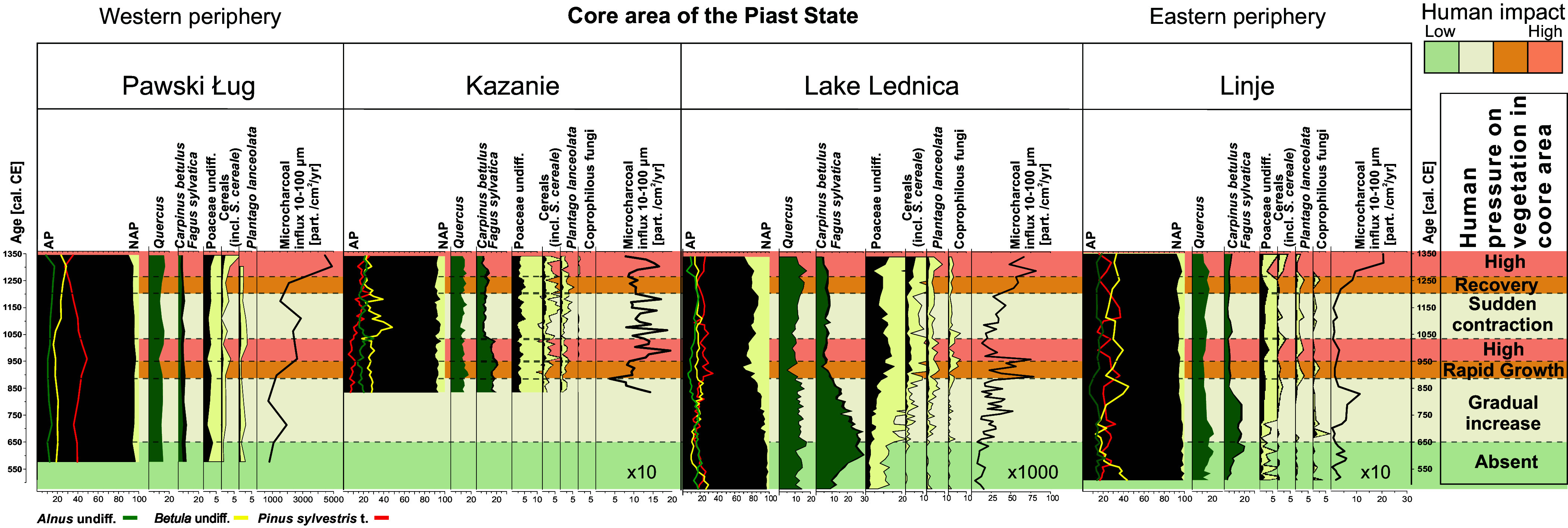
Simplified percentage palynological diagrams from four sites located along south-western to north-eastern direction showing vegetation changes inside the core area and in the periphery of the Piast polity. Selected pollen taxa, composite pollen curves, coprophilous fungi spores, and microscopic charcoal influx are shown. A fivefold exaggeration of some curves was used to better illustrate the effect of a decrease/increase of human impact indicators. Site locations shown in Fig. 3. Lake Lednica data are published here for the first time (full data provided in *SI Appendix*, Fig. S2), the other sites come from already published data (10–12).

**Fig. 3.**
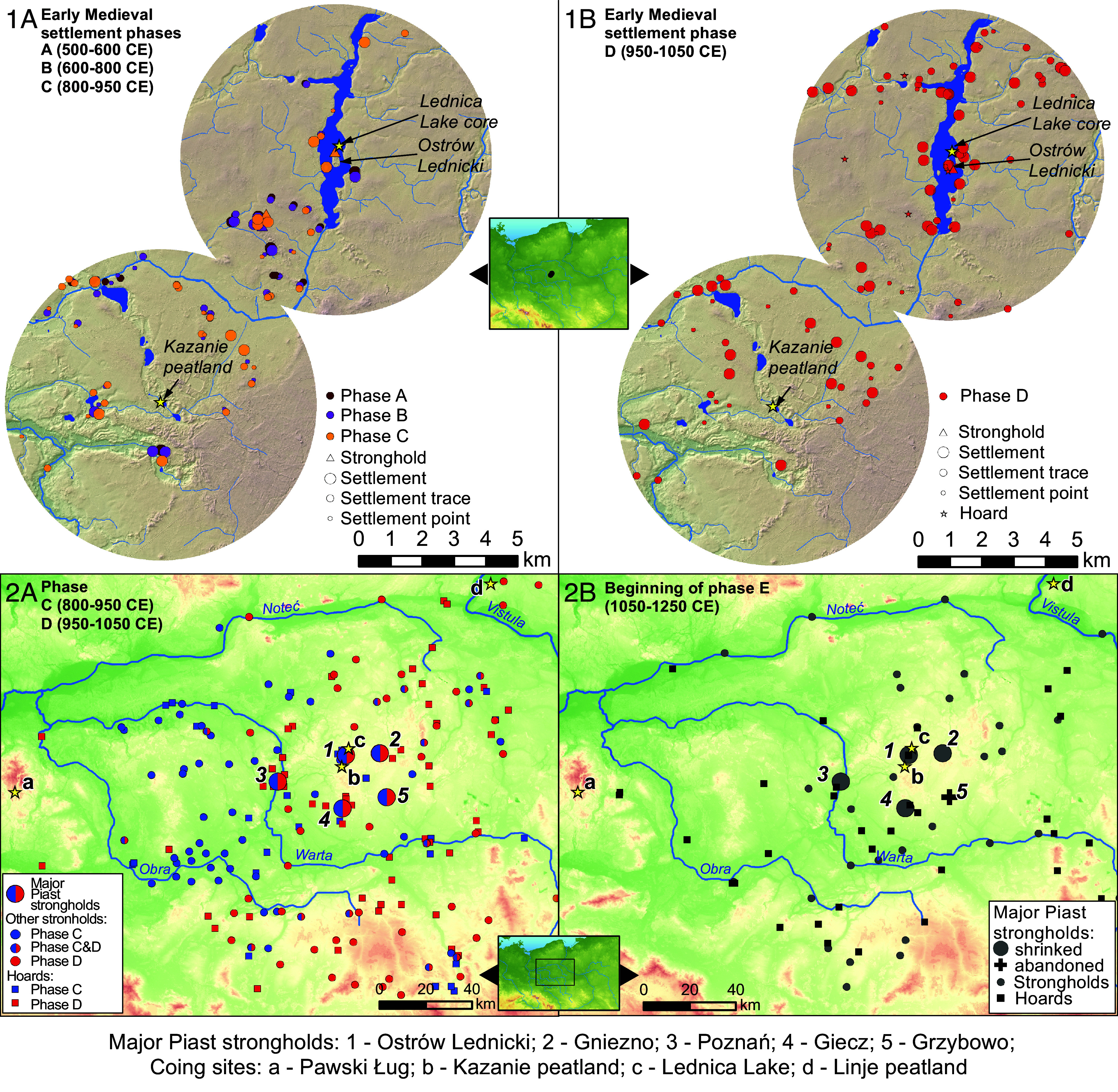
Settlement and economic developments in the Kazanie-Lednica area and the region of Greater Poland in the 6th to 11th c. CE. 1A i 1B: The elevation map of Kazanie peatland and Lednica Lake surroundings was prepared using the LIDAR numerical model obtained from www.geoportal.gov.pl. Each site’s position was defined by rectified and digitized information from the Polish Archaeological Record maps (sheets: 49-31, 49-32, 50-31, 50-32, 51-30, 51-31, 51-32, 52-30, 52-31, 52-32) obtained from www.zabytek.gov.pl. Hoards are based on ref. 13 and provided as Dataset S1, while strongholds have been compiled from a variety of sources (and provided as Dataset S2). The elevation map of Greater Poland was prepared on the elevation products of Shuttle Radar Topography Mission (SRTM) elevation products after www.earthexplorer.usgs.gov.

Four detailed, high-resolution pollen records (one from a lake and three from peatlands) are available for our study (Fig. 2; see Fig. 3 for site locations). In particular, we publish here the pollen record from a sediment core obtained from the bottom of Lake Lednica. Its average resolution is 19 y and its age-depth model is based on 25 Accelerator Mass Spectrometry (AMS) ^14^C dates for the last 1,500 y (*SI Appendix*, Figs. S1 and S2 and Table S1). The coring location is ca. 500 m from the island that served as one of the capitals of the Piast polity. Our second site from the core area of the Piast polity is the Kazanie peatland, situated ca. 9 km SE from Lake Lednica. This site provides robust chronological and sampling control of comparable precision (10). Furthermore, to understand the Piast polity’s impact on the environment outside of its core territory, we discuss here two other high-resolution peat profiles at sites located to the west (Pawski Ług) and the north-east (Linje) of the Lednica-Kazanie area (11, 12).

The Lednica record makes it possible to trace the ecological consequences of significant historical events, starting with the demographic collapse experienced by North-Central Europe during the Migration Period. This demographic collapse occurred as a result of the outward migration of Germanic tribes, in parallel to the fall of the Roman Empire in the south-west in the 5th c. CE. It was followed by a settlement gap of around two centuries and a slow growth of the early Slavic settlement in the area during the 7th to 9th c. CE (5). After the abandonment phase of the Migration Period, the first signs of permanent human settlement are visible around 630 (±27 y) CE in the presence of cereals and ribwort plantain (*Plantago lanceolata*). The oak-hornbeam (*Quercus* sp.—*Carpinus betulus*) forest, naturally regenerated during the Migration Period (the “Absent” phase of human impact in Fig. 2), gradually receded until about 850 (±30 y) CE, as new cultivated areas were established [seen in the slow decrease in arboreal pollen (AP) and the consistent presence of cereal pollen]. The slow decline of hornbeam and the simultaneous slow increase in fire activity may have been caused by selective logging for firewood and agriculture; an asynchronous reduction in hornbeam presence in Central European forests was also observed at other sites, documenting spatial variation in human activity. Hornbeam, growing on fertile soils suitable for agriculture and valued for its high calorific firewood, was among the first species to be extensively cut down. In contrast, oak trees, valued for timber in construction, were less affected by selective logging. This is partly due to oaks being favored in woodland pastures. Therefore, evidence of oak felling around Lake Lednica in the 10th c. CE indicates the rapid transformation of natural forests by the Piast dynasty in the core zone, which is an exception in Polish Lowland area (14).

Thus, in the final decades of the 9th c. and the first half of the 10th c. CE, abrupt landscape change occurred at Lednica. Pollen data reveal deforestation, increase in agricultural activity (rise in cereal pollen), an increase in fire activity (higher sums of microcharcoal), and the development of pasturelands close to the lake (higher meadow and pasture indicators, and spores of coprophilous fungi) (*SI Appendix*, Fig. S2; see also *SI Appendix, Supporting Text S1*). Similar changes, yet of a slightly lower magnitude, occurred at Kazanie: Here, the onset of rapid environmental change occurred in the early 11th c. CE, coinciding with selective logging of hornbeam trees.

At the same time (9th to 11th c. CE), no such drastic changes are visible at the two sites located outside of the core area of the Piast polity, that is Pawski Ług and Linje. To the contrary, a decline in human activity occurred around 1000 CE at Pawski Ług, at the same time as the rapid intensification of human impact transitioned into a stable high state at Lednica-Kazanie. At Linje, selective logging of hornbeam, which occurred simultaneously with an increase in fire activity, particularly in the early 9th c. CE, did not ultimately lead to significant expansion of cultivated areas between 850 and 1050 CE (Fig. 2).

This phase of intensive human impact did not last long. It receded abruptly within less than a century. The cereal cultivation declined at both Lednica and Kazanie around 1040 (±23) and 1045 (±23) CE respectively, with a rapid secondary succession of *Betula* and *Pinus*, much stronger at Kazanie than Lednica. A simultaneous decrease in pastoral activity is visible in the decline of coprophilous fungi and *P. lanceolata* at Lednica. This environmental shift persisted at Lednica for about 170 y, whereas at Kazanie human impact resumed after about 80 y. However, the level of human impact characteristic for the late 10th c. CE was achieved again only in the 14th c. CE, through slow linear growth.

## Settlement Dynamics in the Core Lands.

The Lednica sediment core was obtained from a location close to one of the main centers of power in the Piast polity, the island stronghold of Ostrów Lednicki. Apart from its military purpose, this fortress played an important political, sociocultural, and economic role. These functions were performed thanks to a developed settlement network near the lake, with the population fulfilling servile functions for the Piast ruler (15).

Following the demographic collapse of the Migration Period, settlements only became visible archaeologically in the Lednica region from the 7th/8th c. CE (Phase A-B in Fig. 3, panel 1A). They were probably small and short-lived, functioning for only 2 to 3 generations. Archaeological surveys identified single residential structures in the form of half-timbered houses, as well as farm pits and hearths. These settlement clusters were scattered and usually located near watercourses (13).

In the next period, the so-called prestate period (Phase C, conventionally dated to the 9th c. CE), the settlement pattern became denser and spatially stable, suggestive of territorial communities. Toward the end of this period, local populations constructed their first fortified settlements. These were small single-enclosure structures, located in defensible places at communication nodes. Such fortified settlements were established on the Ostrów Lednicki island at Lake Lednica, and in Moraczewo, at the southern tip of Lake Lednica (16, 17). They were surrounded by small settlement clusters (18). New spaces were settled as well, and settlements increasingly encroached on the upland, while still remaining in the proximity of lakes and rivers. The farming model, based on the alternate use of small plots of land, suggests that at least some of these structures were temporary (13).

Around the middle of the 10th c. CE, the fortification on Ostrów Lednicki was extended, provided with elite residence structures, and connected with the mainland by means of two wooden bridges. Archaeological remains suggest the development of an administrative and treasury apparatus as well as economic facilities to serve this new center of power (8). These activities led to a complete change in the settlement structure in the lake catchment. There was an almost threefold increase in the number of settlements within a period of several dozen years.

Such a rapid population increase could not have resulted solely from natural demographic growth. The area surrounding Lake Lednica became one of the most densely populated regions of Central Europe. It is likely that the Piast rulers resettled communities from the areas they temporarily or permanently conquered, including the region of Pawski Ług where human impact declines at the time when it reaches climactic values at Lednica and Kazanie (Fig. 2). Also, the disappearance of Phase C (800 to 950 CE) strongholds west of the Piast core territory (Fig. 3, panel 2A) may be related to these resettlements. Moreover, the names of at least some of the settlements still existing today, such as Pomarzany, Pomarzanowice, Pomarzanki (“those from Pomerania [a large region to the north of Greater Poland]”), or Czechy (“the Czechs”) suggest foreign or servile origins of their initial inhabitants (15). Additionally, elements of both material and spiritual culture found at some settlements, such as barrow cemeteries similar to those in Pomerania, further support the hypothesis of displacement (19).

It is striking that while the archaeological evidence on the settlement network suggests a densely populated microregion, at the level of 10 inhabitants per square kilometer (13), pollen data suggest levels of cereal farming much lower than in the 16th c. CE, for which a dense settlement network is evidenced in documentary sources (20). The average cereal pollen values at Lednica for the height of the Piast social–ecological expansion are ca 1.5 to 2%—whereas for the 16th c. CE, they reach ca 5 to 7% (these values represent the proportion of cereal pollen within the total pollen sum (TPS) counted in each sample of the sediment core) (*SI Appendix*, Fig. S2). This suggests that despite the agrarian intensification, the Piast polity was not a classical “grain society,” but to a large extent relied on wild ecosystems (forests, lakes) for its nutritional needs, possibly moving some amounts of grain from the outer areas into the core to enhance the provisioning of the elite.

Following the collapse of the Piast polity in the 1030s CE, a notable settlement change occurred. The stronghold situated on the Ostrów Lednicki island was destroyed. Even when the second Piast polity, based in Kraków in south-eastern Poland, regained control over Greater Poland some decades later, the stronghold on Ostrów Lednicki did not regain its earlier significance: Instead, it became the seat of a local administrator. The local settlement network shrank but survived the political collapse. The structures formed in the previous phase have, for the most part, been maintained and even developed over time, during the so-called Phase E, broadly dated to the 11th to 13th c. CE (13, 15). However, when we combine this vaguely dated archaeological record with the more precise pollen records from Lednica and Kazanie (Fig. 2), it is evident that the intensification occurred only in the later part of Phase E, that is the 12th or even 13th c. CE.

## Territorial Dynamics and Military Construction Projects.

Dendrochronological studies confirm and flesh out the picture emerging from settlement surveys and make it possible to follow on the ground the territorial dynamics of the Piast state (expanding over a hundred years from ca 30,000 to ca 400,000 km^2^). They show that the significant elite stronghold at Ostrów Lednicki, with its oldest construction levels dating back to 886 and 912 CE (21), was not isolated. At a similar time, other fortifications were built nearby (Fig. 3): in Giecz (860s CE) (22), Gniezno (ca 940 CE) (8), Poznań (ca 900 CE) (23), and Grzybowo (919-923 CE) (24). Thus, the creation of the Piast core area within eastern Greater Poland involved both ecological intensification and large-scale construction, meant for defense as well as domination. Soon after, the Piast started expanding their territorial base by conquering adjacent regions (Fig. 3). Consequently, while construction projects continued in the core area, similar large fortifications started also to be built outside Greater Poland [e.g., Kruszwica (25), Płock (26), Sandomierz (27), and Głogów (28); see Fig. 1], tightening the Piast grip on the lands they aimed to control. In the later, quasi-imperial phase at the beginning of the 11th c. CE, the Piast engaged in even more ambitious wars of conquests, acquiring far-away regions previously under the control of the neighboring powers of Germany, Bohemia, or Kievan Rus (Fig. 1).

The stronghold of Poznań illustrates these politically motivated military construction projects particularly well, given Poznań’s enormous scale (8). The two small fortifications erected close to each other c. 900 CE were renovated in the 940s to 950s CE when a new horseshoe-shaped rampart was added to the south of them. The two northern fortifications were merged in the 960s CE, and expanded in the following decade with a third section surrounded by a huge wood-and-earth rampart, soon reinforced even further with stone facing. A similar technique was applied to the southern fortification. As a result, Poznań consisted of four fortified sections, thus becoming the most powerful fortification in the Piast polity and perhaps its capital. It withstood a major German military expedition in 1005 CE (29).

The main Piast sites in the core territory continued to play their key role until the political collapse of the Piast polity in the 1030s CE. Written sources report extensive damage to the central Piast strongholds (Gallus and Cosmas in Table 1), but this cannot always be directly corroborated by the archaeological record. While Grzybowo was abandoned (30), the other central settlements survived in a diminished form (29), or were reconstructed after destruction (Ostrów Lednicki; see section above) (Fig. 3, panel 2B).

**Table 1.** Main written sources about the first Piast polity

Source	Place and date of composition [CE]	Genre	Chronological coverage [CE]	Relevance	References
“Gallus Anonymus”	Poland *c*.1112–16	history	10th c.–1110	description of the Piast polity *c*.1090 to 1110; records the memories of several earlier generations	(31)
Thietmar of Merseburg	Saxony 1018	history	919–1018	detailed description of the Piast polity under Boleslav I (992 to 1025)	(32)
Widukind of Corvey	Saxony *c*.968–72	history	919–72	brief mentions of Mieszko I	(33)
Cosmas of Prague	Bohemia *c*.1120	history	9th c.–1125	description of the collapse of the Piast polity in 1039	(34)
*Russian Primary Chronicle*	Kiev, *c*.1115	history	9th c.–1110	occasional mentions of the Piast polity	(35)
al-Mas’udi	Baghdad *c*.956?	geography and history	universal history to 943	tribal geography of central Europe *c*.930	(36) vol. 2, pp. 341–342
Ibrahim ibn Ya’qub	Spain 960s	geography	960s	account of a journey to Prague, description of central Europe	(37), pp. 162–168
rabbinic responsa	Rhineland, *c*.1030	legal cases	late 10th, early 11th c.	trade in the Piast polity	(38)
lives of St Adalbert-Wojciech	Rome *c*.1000	saint’s lives	c.980–997	establishment of the cult of the patron of the Piast polity	(39)
*Dagome iudex*	Rome *c*.990	document	*c*.990	description of the boundaries of the Piast polity	(40)
Bavarian Geographer	Bavaria *c*. 870?	geography	*c*.870	tribal geography of central and eastern Europe	(41)

## Monetary Flows and Exchange Networks.

How to explain the meteoric rise and fall of the Piast polity in Greater Poland? One hint is provided by the exceptionally abundant finds of imported silver coins: A recently published catalog recorded over 80,000 coins mostly coming from c. 200 hoards buried in the 10th and 11th c. CE (42). The coins were mostly imported from the Islamic world, in particular Central Asia, and from Germany, each group accounting for almost 40% of the total. A chronological analysis of the hoards, relying on their termini post quem, i.e., the dates of their most recent coins, allows us to approximate when the coins were imported to Greater Poland.

The concentration of silver hoards in Greater Poland must be considered within the broader context of long-distance trade. Between ca 800 and 980 CE, remarkable quantities of Islamic silver coins (dirhams), perhaps as much as 120 to 240 tons, were imported to northern Europe, particularly Scandinavia, Rus, and Poland (43). Muslim writers report that these coins were acquired by Scandinavian warrior-merchants (also known as Vikings) in exchange for furs and, above all, captives. The large scale of this slave trade is confirmed by frequent mentions of Slavic slaves—as concubines, domestic slaves, bureaucrats, or soldiers—in Muslim sources (44), the prominence of themes of captivity and slavery in northern European sources of this period (45), and archaeological evidence for unusual levels of violence in the Slavic lands, where most slaves in all likelihood originated (46).

Although a latecomer, Greater Poland was one of the main beneficiaries of this system in the final half a century of its functioning (ca 930 to 80 CE), suggesting an active participation of the Piast elite in the long-distance slave trade. After ca 980 CE, when trade with Central Asia was interrupted, the composition of the hoards changed, with German coins replacing the dirhams. Two groups dominate: first, the so-called Otto-Adelheid pennies, and after ca 1040 CE the so-called Kreuzpfennige, both probably struck from silver from the Harz mountains in Saxony. The logic of their inflow has not been fully elucidated (47), but trade in people must have continued until the demand for them decreased ca 1100 CE.

**Fig. 4.**
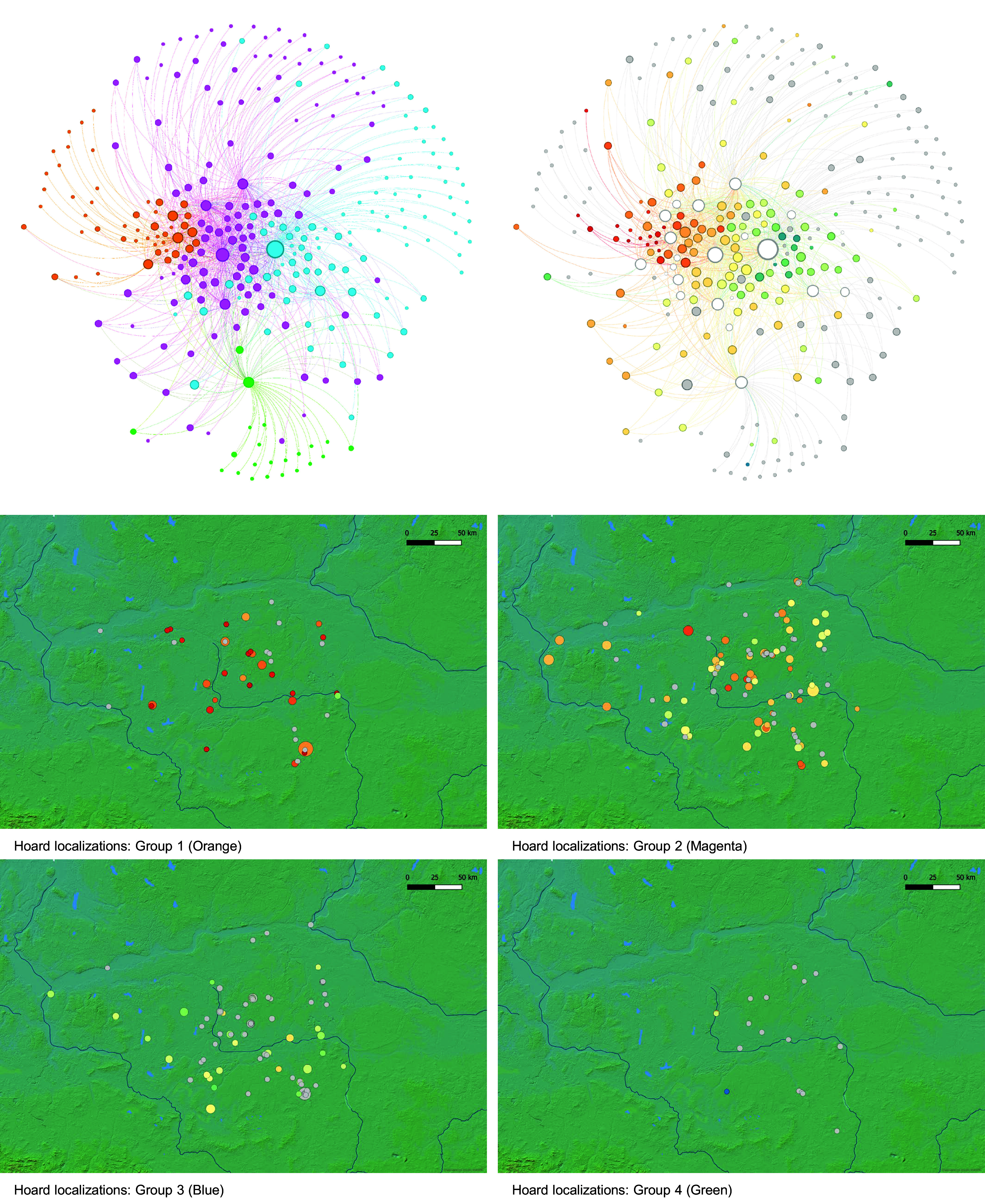
The SNA of silver hoards found in Greater Poland: modularity clustering results (*Top Left*) with overlapping chronology (*Top Right*) together with the geographical distribution of each cluster/group (*Bottom*). The colors of the circles represent hoards’ terminus post quem from the oldest to the youngest (from red through yellow and green to blue; gray for not available). The size of the circles represents the number of coins within each hoard. Figure versions with hoard names are available in *SI Appendix*, Figs. S3–S8.

Our analysis shows that changes in the spatial distribution of the hoards correlate with the evolution of the Piast polity within Greater Poland (Fig. 3, panels 2A and 2B). The earliest hoards, dated before 980 CE, concentrate within its core area and are often found in the vicinity of its main strongholds. This pattern continued in the following decades, which also saw, however, an expansion of the area of hoarding, in particular toward the north and the south. Finally, the political collapse of the 1030s CE is seen in a shift of the hoarding zone away from the old Piast core territory toward the south, in particular the region of Kalisz. To confirm our conclusions drawn from Fig. 3, we performed social network analysis (SNA) on the hoard material (using Dataset S1). The analysis considers the internal composition of every hoard, reflecting the circulation of coins before their deposition. Thus, the basis for grouping is not the dating of hoards, but their level of similarity. The communities identified through the use of Leuven algorithm for modularity clustering form four groups with discernible features. Fig. 4, *Top*, illustrates the gradual shift from the Islamic and Byzantine coins toward German and other Central European coins in the composition of hoards (blue, magenta, orange, and green in Fig. 4 *A*, *Top Left*). The clusters coincide with chronological and geographical changes (note the gradual change in Fig. 4, *Top Right*, from red through yellow and green to blue, representing the dating of the hoards). Hoards from Group 1 (orange), dominated by the Islamic and Byzantine coins, are the oldest and were found mostly in the center of the Piast polity (Fig. 4, *Bottom*). Group 2 (magenta) has the most variable composition with an important component of Bohemian coins. It is the most numerous and is distributed over the whole territory. It can be interpreted as accumulation hoards, composed of coins gathered over an extended period of time. Group 3 (blue), dominated by *Kreuzpfennige*, coincides with the later shift southward. Group 4 (green) contains insufficiently known hoards that often have an admixture of otherwise relatively rare Polish coins. Thus, this analysis supports our earlier interpretation based on Fig. 3.

Silver obtained from long-distance trade was probably used for payments to the military following of the ruler and directly financed the rise of the Piast polity (pp. 162–168 in ref. 37; 48). The emergence of this polity was thus closely related to, and no doubt triggered by, the extension of slave trade networks into central Europe. In order to supply them with captives, the Piasts developed the associated technologies of violence, visible archaeologically but also in the textual record (see below). They thus gained access to unprecedented income and were able to rapidly scale up the agricultural production within their core territory with a coerced and displaced labor force. However, the Piast reliance on these bountiful but unstable exchange networks was also a source of considerable vulnerability for their polity.

## Political Institutions and Ideology.

The Piast polity emerged on the periphery of the Christian and Islamic worlds, and hence relatively quickly entered the “radar” of writers from both of these cultural spheres. Their contemporary yet external descriptions are critical for our understanding of this polity, as the earliest extant local chronicle—the work of an anonymous cleric conventionally known as “Gallus Anonymus” (active in the early 12th c. CE)—relies only on more or less fallible memories of the earliest Piast polity (31). A better-informed witness was Thietmar, bishop of Merseburg in Germany (d. 1018 CE), whose chronicle provides the most detailed information on the early Piast polity (32). Other chronicles from Germany, Bohemia, or Rus supplement further details (33–35), whereas Arabic and Hebrew sources provide insights into the economy of the pivotal 10th century CE (36–38) (Table 1).

None of these sources, however, offers direct explanation for the meteoric rise of the Piast polity. The earliest mentions of its first known ruler, Mieszko I (d. 992), date from the early 960s CE and already reveal a fully functional state: the Spanish diplomat Ibrahim ibn Ya’qub marvels over Mieszko’s powerful army of 3,000 armored warriors and his capacity to pay it regularly in silver (37), whereas the German chronicler Widukind of Corvey calls Mieszko I a “friend of the [German] emperor,” hinting at his involvement in the politics of the German empire (33). This state emerged quite literally from nowhere, as no antecedents can be identified in two tribal geographies dating from ca 870 and ca 930 CE (36, 41).

Written sources are more helpful in their depiction of what we might call the state building processes, in particular their use of Christianity as state ideology and the backbone for the state apparatus. The first two Piast rulers, Mieszko I and Boleslav I (992 to 1025 CE), appear in fact to have consciously imitated the model offered by neighboring German polities, in the first place Saxony. A powerful army gradually moved from raiding to permanently occupying adjacent territories; this evolution was no doubt mirrored in a shift from carrying off the conquered populations to exploiting them on the spot (32–35, 40), attempting to develop a typical military-oriented “grain state” (3), even if wild resources still played an important role. As part of this model, Mieszko I adopted Christianity and had the first bishopric established in the core territory of the state (in Poznan) in the 960 CE. His successor, Boleslav I, supported the Christianizing mission of Adalbert-Wojciech to the neighboring Prussia in 997 CE, where the missionary was killed and thus became a martyr—which in turn boosted Boleslav I’s standing in the international arena of Christian Europe and made it possible to establish state-wide ecclesiastical structures in 1000 CE, including the archbishopric of Gniezno (close to Lednica) (32, 39). This use of Christianity as both ideological and administrative underpinnings of the new state, paired with the extensive fortification-building program described above, shows that the Piast dynasty tried to match territorial expansion with intensive development of networks and connections that would integrate the rapidly growing polity and manage its resource flows. At the same time, Arabic and Hebrew texts reveal glimpses of commercial activities and emphasize the continued importance of the trade in humans (37, 38). Muslim geographers and travelers of the 10th c. CE made a direct connection between the exportation of dirhams to northern Europe and the importation of slaves from there (49). The mere presence of dirhams is thus strongly suggestive of a participation in the slave trade system. While no texts were produced in northern Europe in the 10th c.—making the argument on Piast’s role in this system dependent primarily on the convergence of numismatic and archaeological evidence—written documents from the next century show a continuation of large-scale captive taking in northern Europe and of the reliance of local economies on slave trade and captive labor (e.g., ref. 50).

According to the written sources, in particular the memories preserved in Gallus Anonymus, the collapse of this polity was cataclysmic: Military defeats undermined the prestige of the third ruler, Mieszko II (1025–34), whose demise appears to have been followed by a revolt of the lower strata of the population (31). The final blow was administered by the Czech duke Břetislav I (1034–55) who in 1039 CE ravaged Greater Poland and carried off its population, along with the relics of St. Adalbert-Wojciech (34). The Piast family moved its power base to Krakow and lost control over Lednica and Greater Poland for a couple decades, as dramatically illustrated by tales of wild animals nesting in the ruins of the cathedrals in Gniezno and Poznań (31).

## Discussion: How the Piast Political-Ecological Intensification Failed

**Fig. 5.**
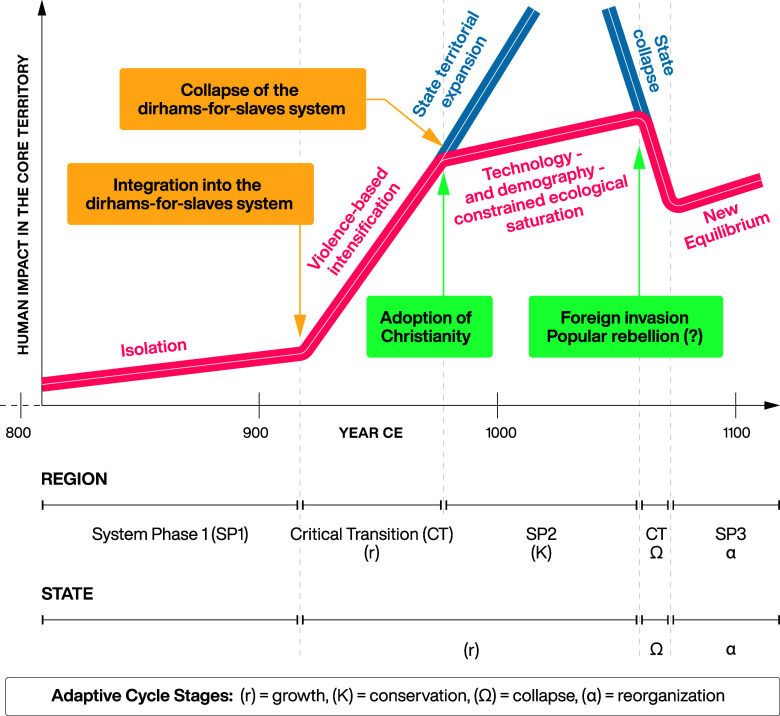
Critical transitions in the Piast system on the regional (Greater Poland) and global (state) scale with relevant critical transition and adaptive (resilience) cycle indications.

The meteoric rise of the new dynasty is well documented in all of our sources and relatively easy to explain as a historical contingency. It was enabled by the inflow of silver in exchange for slaves, and the integration of technologies of violence associated with the acquisition of captives. This initial trigger was followed by social–ecological intensification in the core area, which can be described as a critical transition in the regional social–ecological system (6), accomplished on the regional scale within a time frame of 50 to 70 y (Fig. 5).

On the contrary, the dynasty’s collapse is harder to explain. Essentially, based on the available paleoclimate evidence, we can exclude climate as a factor in this process (*SI Appendix, Supporting Text S2*). Hence, we must look for societal factors, such as the striking disparity between the polity’s social–ecological potential and the scale of its activities. The polity relying on a population in the range of 15 to 30,000 people within its intensively managed inner core (delineated by the strongholds #1-5 in Fig. 3), and further 100 to 150,000 in the large outer core (conquered by ca 960 CE), pursued territorial expansion on a grand scale (Fig. 1; for the population estimates; see *SI Appendix, Supporting Text S3*). This population, however, was too small to sustain constant upscaling of the polity’s operations and the recurring conflicts with its mighty neighbors. Neither were the Piasts able to develop administrative structures needed to control this ever-growing territory, despite their efforts to put in place a church-based administrative-ideological apparatus and construct large strongholds controlling key communication nodes across their territory. Consequently, when faced with military defeats in the 1030s CE, the polity practically disappeared, but what followed was not a wholesale collapse.

The pollen profiles of Kazanie and Lednica are in agreement with one another with regard to a decline in cereal farming and pasturing throughout the region, but they show different trends with regard to forests. This was related to the different degree of human impact on the surrounding forests at the beginning of the 11th century, around Lednica—heavily deforested; around Kazanie—almost untransformed. At Lednica, in parallel to the agricultural decline, there is a slight increase in secondary forest trees (*Betula* and *Pinus*), indicating land abandonment, followed by a slow growth of *Quercus* over the next 150 y (Fig. 2). At Kazanie, the interpretation is more difficult due to the intensive cutting of the natural hornbeam-dominated forest in the decades preceding the agricultural decline. Henceforth, the strong signal of the secondary forest trees (*Betula* and *Pinus*) may be due to the land opening, but even then, it demonstrates that unlike in the preceding century, the new open areas were not used for cultivation, but abandoned (10). In this context, it is striking that the archaeological datasets point to a substantial degree of continuity: the settlement and stronghold networks, while weakened, persist, and the hoarding of silver coins continues, albeit with a different spatial distribution (relative strengthening of the southern periphery of the old core—Fig. 3). Written sources, to the contrary, describe a dramatic, total collapse of the Piast institutions and elites.

While contradictory at the first glance, our sources reveal a multifaceted process of collapse and continuation. The Piast state and its uppermost elite indeed vanished, and this resulted in a sudden end to the Piast ecological intensification. The written sources suggest that the subjugated population openly rejected both the Piast culture of violence and the associated political-religious ideology (i.e., political centralism and Christianity). The collapse occurred within 2 to 4 generations since the forced displacements needed for the social–ecological intensification had taken place, when the memories of the earlier stateless lifeways may have still been vivid—which further facilitated the self-propelled social down-scaling and ecological deintensification as soon as the violence apparatus of the Piasts weakened or disappeared. However, Greater Poland as a region and a social–ecological system did not collapse. Rather, the leap forward it experienced during the Piast ecological-economic acceleration was irreversible and provided the basis for further development throughout the medieval times.

## Conclusions: Broader Insights from the Piast Case Study

Important insights can be gained by looking at the Piast collapse through the lens of the complex systems perspective (Fig. 5). The creation of the initial, tiny political organism in the 900s, not yet even a state, based on intense violence and rapid intensification of resource extraction, put in place a positive feedback cycle of violence-expansion-extraction-(more)violence-(more)expansion-etc. This cycle repeated itself and fueled the dynasty’s expansion. Moreover, in the context of the loose, quasi-anarchic social structures of early medieval Central Europe, there was no preexisting social–ecological mechanism, an established network, or a competing political structure that could slow down this expansion from within. In other words, the positive feedback cycle of violence–extraction–violence spread unabated by any significant negative feedback. However, for a system to reach homeostasis, or dynamic stability, at some point in the process of growth there need to be a balance between the positive (“expanding”) and negative (“withholding”) feedback loops (7). The early Piasts seem to have never reached this point.

Resilience and adaptive cycle theory (51) make it possible to explain the mechanics of this doomed-to-fail process with even more nuance. The adaptive cycle consists of four phases: exploitation/growth—conservation—release/collapse—reorganization/rebound. On the regional scale of Greater Poland, the Piast system achieved some stabilization (conservation) and also the subsequent collapse and rebound phases can be seen further in its history throughout the 11th c. (Fig. 5). More globally, however, the Piast polity collapsed before it even entered the conservation phase: When it ceased to exist in the 1030s CE, it was still in the growth phase (Fig. 5). Crucially, in the adaptive cycle framework, the resilience of a system rests on a balance between accumulated capital (in our case: silver, territory, labor power, agricultural production, etc.) and connectedness (ideological cohesiveness, administrative structures, military control, transportation and exchange networks, etc.). In classical resilience theory, systems can be low in resilience as a consequence of having too many connections built into them. Nonresilient systems are big and rigid, which limits their ability to change flexibly and adapt to new circumstances (this is called hyperconnectivity). In the Piast case, however, the system was not resilient because of underconnectivity: the early Piast state never exited the expansion phase to achieve stability, and hence it never even acquired any significant degree of resilience.

This interpretation is supported by empirical evidence. The Piasts indeed were not in the position to exploit key state-building resources which many other similar attempts relied on. Despite its efforts to quickly create a Christian religious hierarchy, the Piast polity was not able to successfully exploit the cohesive power of religion (unlike in medieval Czechia, Hungary, or Serbia, where the cult of dynastic saints led to a stronger synergy between state-building and Christianization); nor did it develop a good mechanism of co-opting conquered populations and their elites (as Rome and Byzantium were able to do). At the end of the day, the Piast state relied on pure force, as it was created ex nihilo, out of nothing, by local slave traders. Contrary to many successful cases of state building and territorial expansion, in the context of the very thinly populated and largely isolated Early Medieval Central Europe, the Piast elites had no preexisting exchange, cultural, religious, or political networks on which to base their emerging, increasingly complex, political organism; no way of building the necessary minimum of connectivity. Critically constrained by underconnectedness, technology, and demography, they were not able to develop further the ecological intensification they initiated. This lack of reliable, prior networks on which to base their state-building efforts, proved a fatal liability that revealed itself in the rapid collapse of the first Polish state—despite the Piasts’ efforts to develop such networks of their own. In the terminology of the adaptive cycle and complex systems theories, because of the lack of negative feedbacks within the Piast system, the new polity was accumulating capital much faster than it was capable of stabilizing it through increasing its own connectedness.

While we can offer this system-level explanation of the phenomena that are visible in our empirical material, the available evidence does not provide enough information to trace individual or group human agency (such as in refs. 52–54) in these social–ecological processes. While one of our written sources (31) hints at a possibility of a popular rebellion, perhaps even driven by the rejection of the Piast Christian ideology, neither archaeology nor paleoecology can corroborate this claim (55) that comes from a text written with a strong political bias four generations after the events—even if paleoecology in particular reveals the actual scale and the fast pace of the collapse. Active popular rejection of the Piast rule in the core territory of Greater Poland thus remains just one of several equifinal explanations, with the other ones including the invasion and plundering done by Břetislav I or the global social–ecological inefficiency of the Piast system. Thus, in agreement with complexity science, while we cannot easily scale our analysis down to the level of individual agents or local subsystems, we are still able to see the key emergent phenomena and their trajectory.

To conclude, the Piast case study we present here is unique for the multifaceted, even if fragmented, character of its documentation. It allows deep insights into the ecological, economic, political, religious, and cultural dimensions of the rise-and-fall mechanism of the early states. The Piast story can thus serve as a comparison, if not a model, for similar social–ecological phenomena in other parts of the world and in other historical periods: such as, for instance, the emergence of slaving states in sub-Saharan Africa in the medieval and early modern periods (56). Here and there, external demand for slaves created conditions for local state formation in areas determined less by environmental conditions (the soils in Greater Poland are rather poor) than by the key slave trading routes. The Piast model invites questions about the ways in which the elites of such newly formed states attempted to stabilize their political structures (despite the destabilizing social effects of the slave extraction), and in particular the role of social–ecological intensification in such attempts.

More indirectly, the Piasts provide comparative material for those early states for which detailed written or ecological data are lacking. For instance, the sudden rise of Cahokia, a polity that formed in the American Bottom (a large floodplain on central Mississippi) in the 11th to 12th c. CE, was also accompanied by forced ecological intensification, in that case focused solely on grain (maize) (57), while the Piasts attempted to boost grain production while still relying on wild resources. Cahokia’s population size was of the same order of magnitude as that of the Piast state (30 to 40,000 inhabitants) and it also dissolved in the context of a social conflict (58, 59). Both collapse stories also invite questions about the labile role of religious ideology in stabilizing these polities, with some evidence in the Piast case of the ideology’s violent rejection by the nonelite population, followed by its partial dispersal.

In brief, the Piast case can serve as an exceptionally well-documented model of the state-formation “package,” consisting of economic triggers, new technologies of violence, ecological intensification, coercive labor, and religion-based state ideology. At the same time, it provides a nuanced framework for understanding collapse and exemplifies the distinction between an elite collapse i.e., disappearance of a political superstructure based on coercion, and broader societal continuity. Crucially, this in-depth understanding of the acceleration and collapse processes would not have been achieved without the holistic combination of high-quality environmental, archaeological, and written evidence.

## Materials and Methods

### The Age-Depth Model of Lake Lednica.

Chronology is based on plant material selected from the lake sediments. 37 plant terrestrial macrofossil samples (mostly epiderms) spanning the top 245 cm of sediment were dated with the radiocarbon (^14^C) method AMS in the Laboratory of Ion Beam Physics, ETH (34 dates) and at Poznan Radiocarbon Laboratory (Poland, lab. code–Poz) (three dates) (*SI Appendix*, Table S1). The samples were treated with Acid Base Acid method to remove contamination (60, 61). Clean samples were then freeze-dried, weighed, and packed into Al cups for combustion in Elemental Analyzer. The samples contained less than 100 μg carbon therefore the CO_2_ from the combustion was directed to the gas ion source for Gas Ion Source Accelerator Mass Spectrometry (GIS AMS) analysis (62) using the Mini Carbon Dating System (MICADAS) at the ETH Zurich facility (63). The absolute chronology was constructed using the OxCal v. 4.4.4 software (64), applying the *P_Sequence* function, the IntCal20 (65). Seven samples were excluded from the model and treated as outliers, as indicated in *SI Appendix*, Table S1. For better article readability, the age is presented as a µ (mean) value of the modeled age, rounded to five. As the entire period was within the last 2000 y, each date is implicitly CE.

### Pollen and Charcoal Analysis of Lake Lednica.

For the analysis of pollen in Lake Lednica, described in this study, we used a total of 61 samples, each with a 1 cm^3^ volume. Other palynological and microcharcoal data presented in Fig. 2 are sourced from previously published studies (10–12).

Samples were treated with 10% hydrochloric acid (HCl) to dissolve carbonates, heated in 10% potassium hydroxide (KOH) to remove humic compounds, and finally soaked in 40% hydrofluoric acid (HF) for at least 24 h to remove the mineral fraction. It was followed by acetolysis (66). One *Lycopodium* tablet (14,285 spores; produced by Lund University) was added to the samples (67). Sample slides were counted under a binocular microscope until the TPS at least 500 AP grains. Palynomorphs were analyzed under a binocular microscope until the TPS in each sample exceeded 500. Pollen taxa were identified using atlases and keys (68, 69). Nonpollen palynomorphs, including fungi and algae, were also counted, and their identification was facilitated by the Non-Pollen Palynomorph Image Database (70). The calculation of pollen percentages followed the formula: taxon percentages = (number of taxon grains/TPS) × 100%, where TPS include AP and NAP taxa, while excluding aquatic and wetland plants, including Cyperaceae, and cryptogams.

Microscopic charcoal particles, exceeding 10 μm in size, were counted on pollen samples (71, 72) until the cumulative count of charcoal particles and *Lycopodium* spores exceeded 200. For macroscopic charcoal analysis, 61 samples (sample size: 1 cm^3^) were prepared by wet sieving the peat through a 500-µm mesh. Particles >600 µm were then counted under the stereoscopic microscope; particles of this size represent local fire activity (73). Microscopic and macroscopic charcoal influx or accumulation rates (respectively, particles/cm^2^/year) were calculated using the charcoal concentrations and Pollen Accumulation Rates (71).

### Settlement Dynamics Analysis in the Area of Lednica Lake and Kazanie Peatland.

The Early Medieval settlement view around the coring sites was prepared/drawn within a circle of 5 km radius according to the site-catchment analysis standard for agricultural societies (74). The archaeological sites position was redrawn from 10 sheets (1:25,000) of Polish Archaeological Record (PAR) mapping programme, marking five types of sites: strongholds, settlements, settlement traces, settlement points, and hoards. Each entity (site) was assigned with chronological information after the PAR explanation list. We have included four phases: A, B, C, and D grouped into two clusters: ABC and D (75).

### SNA of Coinage Data.

SNA has been performed on the corpus of hoards found in Greater Poland with the use of GEPHI Graph Visualization and Manipulation software (76; version: 0.9.7 202208031831). Whenever possible, a hoard has been ascribed with the terminus post quem (tpq) with decadal accuracy spanning from 930 to 1140 CE. The identifiable coins in the hoards have been assigned to the following categories: Bohemian, Byzantine, Danish, English, French, German, Hungarian, Imitations, Indian, Irish, Islamic, Italian, Kreuzpfennige, Moravian, Norwegian, Persian, Polish, Roman, Russian, Scandinavian, Swedish, Turkmen. Nodes in the network are defined as either a hoard or a category. Edges are defined as the relation between a hoard and the category with the number of identified coins within a given category being the weight of the edge. The outsized hoards such as Dzierżnica II have been excluded from the analysis as outliers. The communities within the network have been identified with the use of modularity clustering at the resolution of 1.0 (77) as a way to detect the groupings of hoards with similar composition.

The resulting network has been visualized with the use of Force Atlas gravity-based visualization algorithm. The linked nodes gravitate toward each other, while more distant ones push each other away, until the whole network achieves equilibrium, thus the geometric distance between nodes reflects their relative similarity. The size of a node represents the number of coins assigned to it.

Graph 1: The nodes representing hoards have black labels and varying colors, while those representing categories are white with red labels. The color of a hoard-node represents its tpq from the oldest to the youngest (reds through yellows to greens; gray for not available).

Graph 2: The color of a node represents the community to which it has been assigned (Group 1 – blue, Group 2 – pink, Group 3 – orange, Group 4 – green).

In the next step, the hoards have been divided according to the identified communities and plotted on maps. For plotting the resulting graphs on maps, the QGIS Geographic Information System, version: 3.4.5-Madeira (78) and the mapping resources the Environmental Systems Research Institute have been used.

## Supplementary Material

Appendix 01 (PDF)

Dataset S01 (XLSX)

Dataset S02 (XLSX)

## Data Availability

All study data are included in the article and/or supporting information. Previously published data were used for this Fig. 1 work. Fig. 2 uses data published previously in other journals–all information is provided in the references in the figure caption. From Fig. 1 caption: Based on ref. 8, p. 254, and ref. 9, p. 65.
