# Supplementary material for: Unbalanced social–ecological acceleration led to state formation failure in early medieval Poland
Source: Proc Natl Acad Sci U S A. 2025 Apr 21;122(18):e2409056122. doi: 10.1073/pnas.2409056122 (PMC12067273; doi:10.1073/pnas.2409056122)
Supplement: Supplementary file 1 — Appendix 01 (PDF) [file pnas.2409056122.sapp.pdf]

PNAS

Supporting Information for

Unbalanced social–ecological acceleration led to state formation failure in early medieval Poland

Adam Izdebski[1,2,3], Sambor Czerwiński[4], Marek Jankowiak[2,3], Marcin Danielewski[5], Sabina Fiołna[6], Raphael Gromig[1,7,8], Piotr Guzowski[9], Negar Haghipour[10,11], Irka Hajdas[10], Piotr Kołaczek[12], Mariusz Lamentowicz[12], Katarzyna Marcisz[12], Jakub Niebieszczański[5], Paweł Sankiewicz[13], Bernd Wagner[8]

[1] Palaeo-Science and History Group, Max Planck Institute of Geoanthropology, Jena 07743, Germany
[2] Faculty of Liberal Arts, University of Warsaw, Warsaw 00-927, Poland
[3] Centre for Systemic Risk Analysis, University of Warsaw, Warsaw 00-927, Poland
[4] Department of Geomorphology and Quarternary Geology, Faculty of Oceanography and Geography, University of Gdańsk, Gdańsk 81-378, Poland
[5] Faculty of Archaeology, Adam Mickiewicz University in Poznań, Poznań 61-614, Poland
[6] Department of Philosophy and Humanities, Institute of Greek and Latin Languages and Literatures, Freie Universität Berlin, Berlin 14195, Germany
[7] Department of Earth Sciences, Simon Fraser University, Burnaby V5A 1S6, BC , Canada
[8] Institute of Geology and Mineralogy, University of Cologne, Cologne 50674, Germany
[9] Faculty of History, University of Bialystok, Białystok 15-403, Poland
[10] Laboratory of Ion Beam Physics, ETH Zurich, Zurich 8093, Switzerland
[11] Earth Sciences Department, ETH Zurich, Zurich 8093, Switzerland
[12] Climate Change Ecology Research Unit, Adam Mickiewicz University, Poznań 61-680, Poland
[13] Museum of the First Piasts at Lednica, Lednogóra 62-261, Poland

Corresponding Author: Adam Izdebski
Email: izdebski@gea.mpg.de

This PDF file includes:

Supporting text S1 to S3
Figures S1 to S10
Table S1
Legends for Datasets S1 to S2
SI References

Other supporting materials for this manuscript include the following:

Datasets S1 to S2

Supporting Information Text S1

Description of the most important man-made vegetation changes at the Lake Lednica site

While the Lake Lednica vicinity (especially the region between Gniezno and Poznań) has been extensively studied regarding vegetation changes and identified changes in human impact during the Middle Ages, we previously lacked the reliable sampling resolution and chronology to place these findings on a time scale (1-5).

Therefore, we decided to conduct new, high-resolution paleopalynogical research in the region, crucial to understanding the processes involved in the transformation from egalitarian to state structures and trace the most important ‘ups and downs’ of economic activity and their paleoenvironmental signal.

Paleoecological analyses performed in high or even continuous sampling (each sample is vertically contiguous) reduce the likelihood of a suck-in and smear type of interpretive error (6). This involves, among other things, assuming a link between parallel changes (6-8). With greater distances between samples, there is an increased likelihood of missing short-lived natural phenomena recorded in narrow layers of sediments and peats. This, combined with the use of chronologies based on low-resolution dating, can lead to erroneous attribution of natural changes to historical events. Hence, the use of high sampling and chronological resolution enables reliable interpretation and placement on the time scale of the results of paleoecological analyses. With a precise palaeorecord based on palynology supported by archaeological and historical evidence, we were able, for the first time, to accurately place human impact on a time scale, and to assess the environmental impact of humans in this historic region.

The palynological analysis of Lake Lednica has revealed typical patterns associated with forest regeneration and deforestation in this part of Central Europe (9-10), but these have not previously been precisely placed on a time scale, which seems crucial for capturing past human impact. A recent study highlighted that fluctuations in hornbeam pollen percentages can be indicators of local human pressure on the environment (9). Our observation at Lake Lednica reveals that the hornbeam regeneration phase occurred between 500 and 620 CE. Over 120 years, hornbeam pollen percentages experienced a notable 20% increase, peaking and maintaining optimal ecological conditions for an additional 80 years (until approximately 700 CE).

Throughout this period, the marginal presence of cereal pollen signifies a cessation of human influence in the vicinity of the site or the complete abandonment of the area. These conditions allowed for the secondary succession of forest, predominantly dominated by hornbeam, on land previously used for agriculture. Evidence supporting the intensive agricultural transformation of the area before 500 CE, along with corroborating palynological data from the region (1-5, 11-13), is evident in the diminishing proportions of ruderal pollen in the 6th and 7th centuries CE.

Palynological data emerges as a crucial tool for confirming demographic collapse, indicative of a pronounced reduction in human pressure on the environment that led to forest regeneration, probably due to migrations during the Migration Period (14-15). Despite recent progress in understanding environmental consequences during and after the Migration Period (9, 16), precise spatial and temporal patterns remain inadequately elucidated. Addressing this knowledge gap necessitates both local and regional high-resolution palynological investigations, in tandem with archaeological investigations.

Between 620 and 700 CE, a period characterized by weak human impact, subtle increases in the percentages of various human impact indicators (e.g., cereals, ruderals vegetation, microcharcoal influx, and notably Poaceae) are observed near the core collection site. These indicators reflect minor-scale agricultural practices that did not substantially influence the population of hornbeam and other deciduous trees, such as linden. Additionally, there is a notable rise in *Fraxinus excelsior* percentages during this period, signaling the regeneration of periodically flooded forests (17). An increase in *Cannabis*/*Humulus* pollen is also recorded during this period. On the one hand, common hops are widespread in riparian forests, which may reflect the regeneration of these vegetation. However, the temporal trends of this taxon's curve, exhibiting increases during periods of high human impact and declines during phases of low human activity, imply a significant association with human factors.

From 700 to 840 CE, an increase in Arboreal Pollen (AP) and a gradual decline in hornbeam pollen confirm the selective clearing of hornbeam, linked to the occupation of fertile soils suitable for agriculture where this species thrived. The preference for hornbeam wood, attributed to its high calorific properties and challenging processing (18), likely led to its prioritized logging. Indirectly, the economic use of hornbeam wood may also be evidenced by the increasing influx of microcharcoal during this period. Such palaeoecological patterns (hornbeam decline and simultaneous increase in microcharcoal influx) are present at other sites from the Polish Lowlands (9), including those reanalyzed in this article Kazanie site (19).

Short-term declines in the percentages of cultivated pollen between 700-840 CE can be associated with intensive soil use and subsequent exhaustion, prompting a shift in cultivation to areas slightly farther from the core collection site. This shift is reflected in the palynological data, signaling a reduced agricultural impact on the landscape. During these periods, increases in pioneer taxa, such as birch and pine, are noted, indicating temporary abandonment and overgrowth of some areas around the study site. This situation is evident between 730-780 CE and 810-840 CE. Moreover, a decrease in ruderal pollen percentages is also observed during these phases.

The determination of the precise timing of the direct colonization of Ostrów Lednicki Island poses a challenge based on palynological data. The progressive deforestation and human economic activities since the early 7th century CE makes it challenging to pinpoint the exact initiation of island settlement. Some societies may have already established themselves on the island by the early 8th century CE. Indications of this process are suggested by the distinct decline in AP during 700-725 CE, signifying potential deforestation on the island or its immediate vicinity. This is corroborated by a distinct reduction in the pollen percentages of trees growing near water, such as *Alnus* or *Salix*, which suggested the clearing of the lake shore zone.

The environmental transformation process in the Lednica Lake area, spanning over 200 years from the 7th century CE, notably intensifies between 860 to 1040 CE. This acceleration is evident through an increasingly rapid decline in AP and a higher proportion of cultivated land in the landscape, along with expanded ruderal areas. A simultaneous rise in coprophilous fungi indicates grazing of herbivores (most probably livestock), likely on the island (20). The most significant environmental transformations attributed to heightened human activity around the site have emerged since 910 CE. This is characterized by intensive deforestation of deciduous species, including the disappearance of certain trees (*Tilia cordata*, *Fraxinus excelsior*).

The significant decline in oak's proportion at the beginning of 10th century CE is attributed to its historical economic use in constructing various buildings, particularly in fortified settlements (21-22). Its exceptional physical and mechanical properties, including rot resistance and hardness, made it highly valuable in a landscape abundant with wetlands and lakes. The demand for oak during the construction of fortified settlements likely led to extensive cutting. While the construction of bridges in 963-964 CE, requiring substantial oak wood, is less evident in the paleorecord, this discrepancy could be attributed to radiocarbon dating errors or deforestation from neighboring regions.

The landscape witnesses increased agricultural use, highlighted by increased proportions of cultivated land pollen indicators. The signs of livestock husbandry can be inferred from rising values of meadows and pastures pollen indicators, especially *Plantago lanceolata*, and the presence of coprophilous fungi spores. Human activity also induces cultural eutrophication of the reservoir, manifested by higher shares of *Pediastrum* algae (23), particularly between 890-990 CE. This period records peak values in microcharcoal accumulation rates, indirectly indicating increased human economic activity.

The 11th-century state crisis and Bretislav's invasion in 1039 CE led to extensive destruction, depopulation, and subsequent landscape rewilding. This is evident for example in the increase in AP pollen over the following 200 years. Demographic effects of the crisis are reflected in a marked decline in cereal and ruderal pollen shares between 1050-1210 and 1050-1100 CE, respectively.

Supporting Information Text S2

Paleoclimate evidence for Central Europe in the 10th-11th c. CE

The scholarship on the history of climate and society in Poland and Central Europe more broadly is relatively abundant and makes it possible to identify the types of climate anomalies and weather extremes that had the potential to push a pre-industrial agricultural society in this part of Europe into crisis. Potential climatic stress factors can be related to precipitation and temperature. Concerning precipitation, a dry summer could lead to harvest failures, yet the impact of abnormally wet springs or summers could be even more dramatic (24). As far as the temperature is concerned, the most significant risk factor was the winter temperature; if very low, this could lead to negative health consequences for the population and increased mortality (25).

Summer hydroclimatic conditions in the region of the first Piast polity can be reconstructed thanks to the Old World Drought Atlas (OWDA) (26). Figures S9-S10 (above) were generated from the OWDA and show the June-July-August drought index (PDSI) for the region of Greater Poland (the Piast inner core territory). As is visible from the figures, while the Piast state developed under slightly increased aridity, there was no extreme drought or any sudden reversal of trend in the decades of the political crisis, the 1020s and 1030s CE. There is no evidence of increased wetness during this period. On the contrary, there is abundant evidence from cores extracted from several wetlands in Central and Northern Poland, indicating stable hydroclimatic conditions over this period (27-29). To conclude, the first Piast state did not experience any hydroclimatic stress.

Summer temperature conditions for Central Europe in the 11th c. can be reconstructed with relative certainty thanks to dendroclimatic data. The Alpine summer temperature reconstruction shows no significant anomaly, greater than ca 1℃, during the first half of the 11th c. CE (30, see also 31). The relevance of this trend to the Polish lands is further confirmed by the later agreement of this trend with the trend visible in the Central European temperature reconstruction from the Tatra mountains (which started in 1040 CE) (32). As regards the winter temperature conditions, in the period of 990-1040 CE (50 years prior to the “Piast collapse”) there was no major northern hemisphere volcanic eruption (33) that would have caused winter cooling effects across Europe similar to those of the Eldgjá eruption (Iceland) in 939 CE (34).

To conclude, ample paleoclimatic evidence exists for the 50 years preceding the “Piast collapse” of the 1030s CE that excludes the occurrence of biophysical stress strong enough to have caused major disruption to this society’s health or subsistence.

Supporting Information Text S3

Demographic estimates for the Piast core territories, c. 930 and 960 CE

At the outset, around 930-940 CE, the Piasts controlled a tiny territory of ca 3,000 km^2, delineated by the five major strongholds shown on Fig. 2 (strongholds #1-5). There is no direct information on the extent of land under cultivation within this total area. However, historical documentation that survives from the 16th c. CE, the climactic point in the development of pre-industrial agriculture in Poland, shows that under this maximum agrarian intensification, the arable land amounted to ca 20% of the entire territory of the region in question (35). In the 10th-11th c. CE, even at the height of the Piast social-ecological intensification, this ratio must have been much lower: the average cereal pollen values at Lednica during the height of the Piast social-ecological expansion are ca 1.5-2% – whereas during the 16th c. CE, they reached ca 5-7% (Fig. S2). However, to account for the lesser importance of grain in early medieval Central European diets than in the 16th c., we consider below two scenarios of annual grain consumption per person.

We assume 10% of total land being under cultivation in the Piast core territory, which amounts to 30,000 ha. In medieval times, a typical Polish single-raddle farm in a two-field system operated on some 16.8 ha (36). Theoretically, there could have been 1,785 standard medieval farms in the core Piast territory in the 10th c. CE (please note: we do not know what the actual agrarian regime at that time was, whether individual farms or large estates based on servile labor or else, but we use

this information to approximate probable population and production levels). Assuming production levels at 2.5 grain harvested from one grain sown, a standard farm would have produced 1.937 tons of grain per year, which would amount to the total production of the Piast core territory in the range of 3,500 tones. If we take 0.25 ton as the annual grain consumption per person (documented for medieval and early modern Poland (37)), we arrive at the carrying capacity of the Piast core territory under the 10th c. CE conditions in the range of ca 14,000 people. Of these, if each farm would have been worked on average by 5.5 people (38), some 4,000 people did not have to work the land. However, if we reduce - which is plausible - grain consumption by 50%, we arrive at the estimate of ca 30,000 people. This range of 15-30,000 inhabitants in total, of which a few thousands non-producing population, would agree with the estimates of the early Piast military personnel (a few hundred people), which would have been accompanied by families and slaves, plus the craftsmen and a tiny managerial group. However, to sustain these levels of non-producing population, the military elite would have to be able to extract all surplus grain from the producing population. This means that already the early Piast state was at the limits of its ecological-demographic sustainability (and please note we employed rather optimistic coefficients in our estimations).

Critically, just a few decades later, in ca 960 CE, the written sources already (plausibly) state that the military personnel under the Piast command amounted to ca 3,000 warriors (39). To feed this number of warriors together with their slaves and families, in addition to the emerging religious-administrative elite plus the necessary craftsmanship supporting elite and military consumption, it was necessary to reach several other areas that surrounded the initial core territory, and exert tight political control over them. It should be noted, however, that few if any such areas had the population density of the core Piast territory. If we assume a ten-fold increase in the territory size, but a smaller land under cultivation ratio (3%), we obtain the cultivated area of ca 90,000 ha, three times the size of what was available in the Piast core territory itself. With these numbers, likewise under tight military control of the producing population, it may have still been possible to feed and maintain 3,000 warriors together with all the other non-producing population (hence ca 10-12,000 non-producing population).

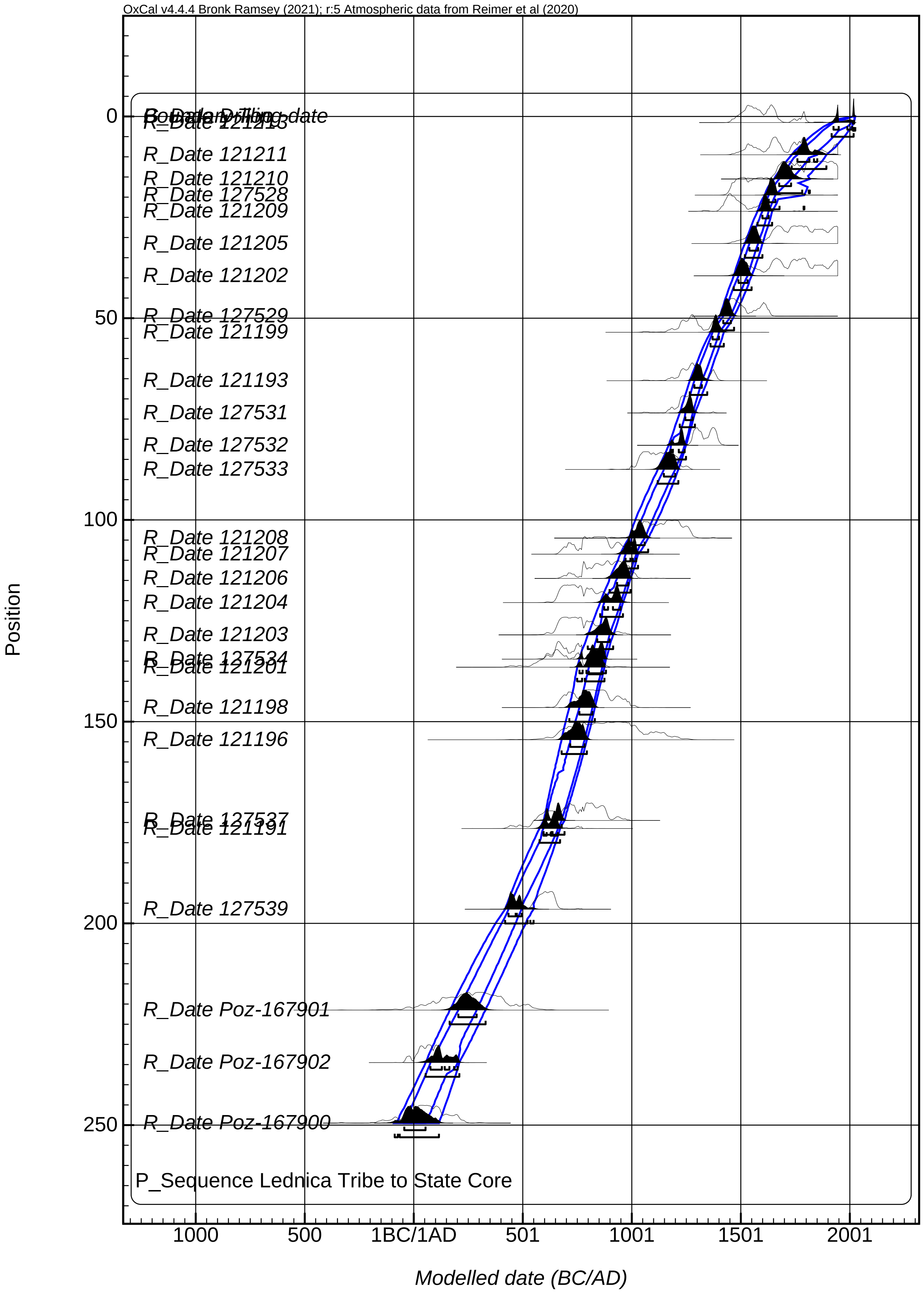

OxCal v4.4.4 Bronk Ramsey (2021); r:5 Atmospheric data from Reimer et al (2020)
Drilling date
R_Date 121213
R_Date 121211
R_Date 121210
R_Date 127528
R_Date 121209
R_Date 121205
R_Date 121202
R_Date 127529
R_Date 121199
R_Date 121193
R_Date 127531
R_Date 127532
R_Date 127533
R_Date 121208
R_Date 121207
R_Date 121206
R_Date 121204
R_Date 121203
R_Date 127534
R_Date 121201
R_Date 121198
R_Date 121196
R_Date 127537
R_Date 121191
R_Date 127539
R_Date Poz-167901
R_Date Poz-167902
R_Date Poz-167900
P_Sequence Lednica Tribe to State Core
Position
0
50
100
150
200
250
1000
500
1BC/1AD
501
1001
1501
2001
Modelled date (BC/AD)

Fig. S1. Age-depth model of the Lake Lednica Core based on 30 radiocarbon ages,was constructed using the deposition model of OxCal 4.4 (40) and the INTCAL20 calibration curve (41).

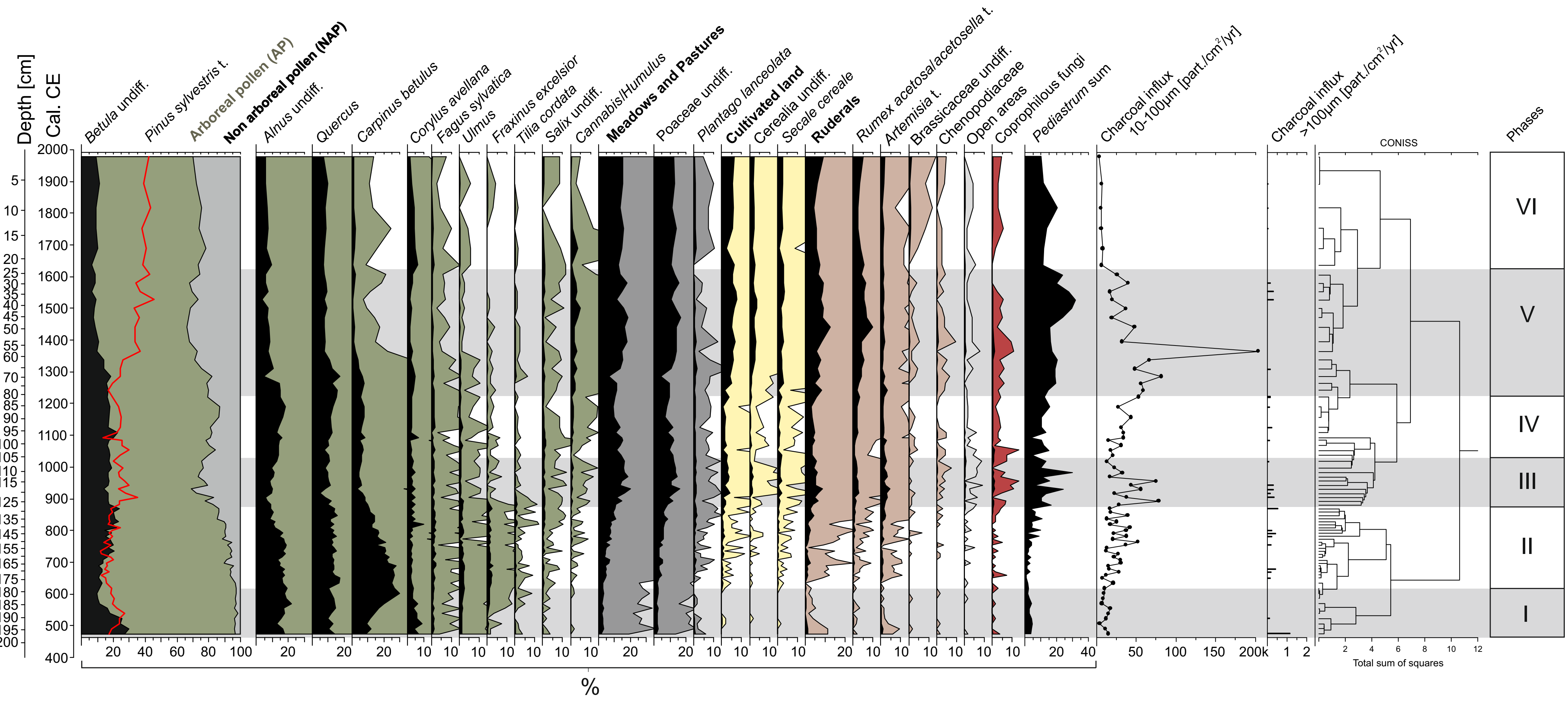

Depth [cm]
Cal. CE
Betula undiff.
Pinus sylvestris t.
Arboreal pollen (AP)
Non arboreal pollen (NAP)
Alnus undiff.
Quercus
Carpinus betulus
Corylus avellana
Fagus sylvatica
Ulmus
Fraxinus excelsior
Tilia cordata
Salix undiff.
Cannabis/Humulus
Meadows and Pastures
Poaceae undiff.
Plantago lanceolata
Cultivated land
Cerealia undiff.
Secale cereale
Ruderals
Rumex acetosa/acetosella t.
Artemisia t.
Brassicaceae undiff.
Chenopodiaceae
Open areas
Coprophilous fungi
Pediastrum sum
Charcoal influx 10-100µm [part./cm²/yr]
Charcoal influx >100µm [part./cm²/yr]
CONISS
Phases
VI
V
IV
III
II
I
%
Total sum of squares

Fig. S2. Extended percentage palynological diagram from Lake Lednica. Selected pollen taxa and major indicators, coprophilous fungi spores, *Pediastrum* algae sum and micro- and macroscopic charcoal influx are shown. A tenfold exaggeration of some curves was used to better illustrate the effect of a decrease/increase of human impact indicators. The phases are defined based on the CONISS algorithm in Tilia software to describe the general changes within the pollen assemblages (42).

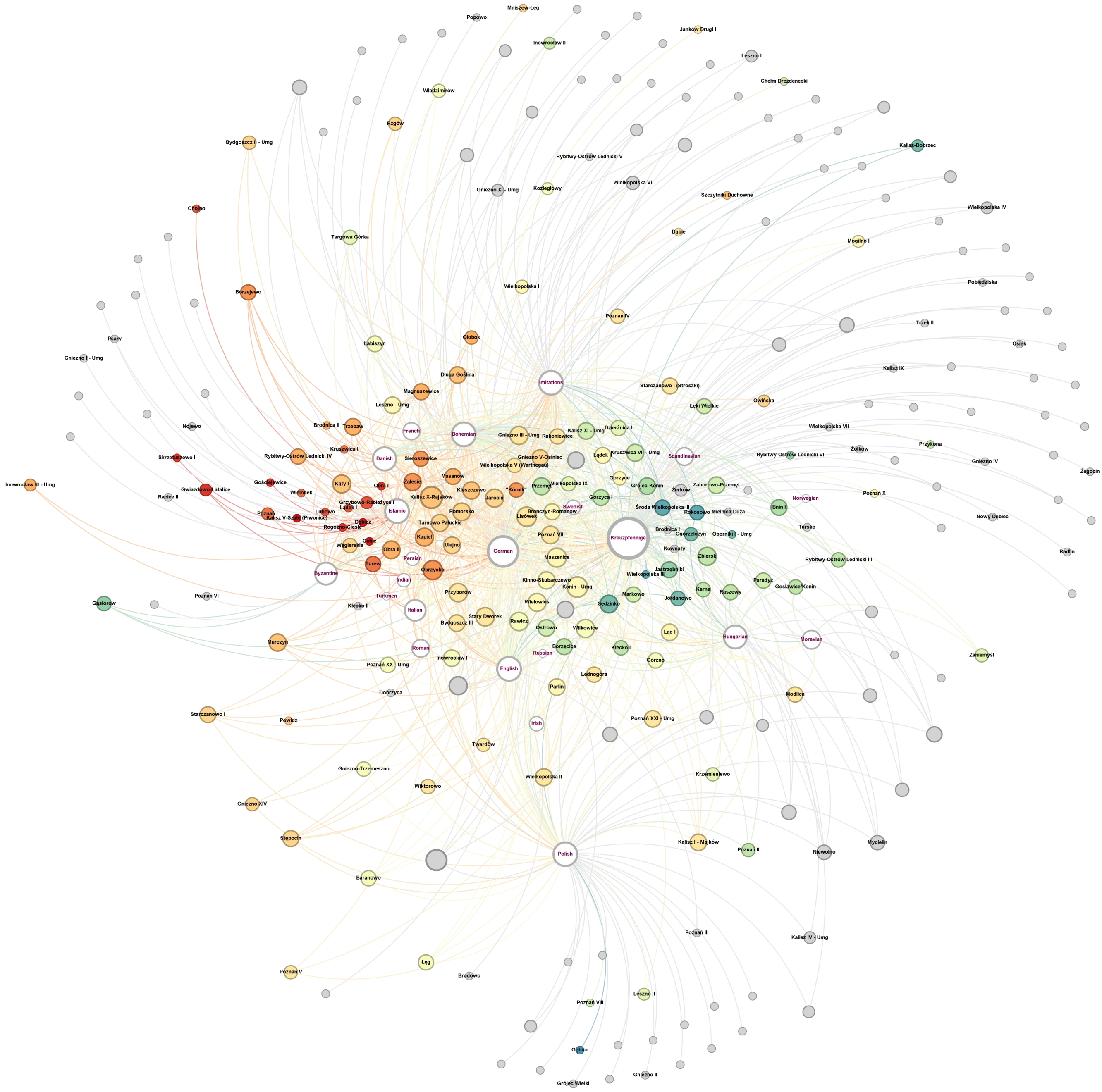

Fig. S3. The graph visualization of the social network analysis of silver hoards found in Greater Poland. Graph 1: The nodes representing hoards have black labels and varying colors, while those representing categories are white with red labels. The color of a hoard-node represents its tpq from the oldest to the youngest (reds through yellows, greens to blue; gray for not available)

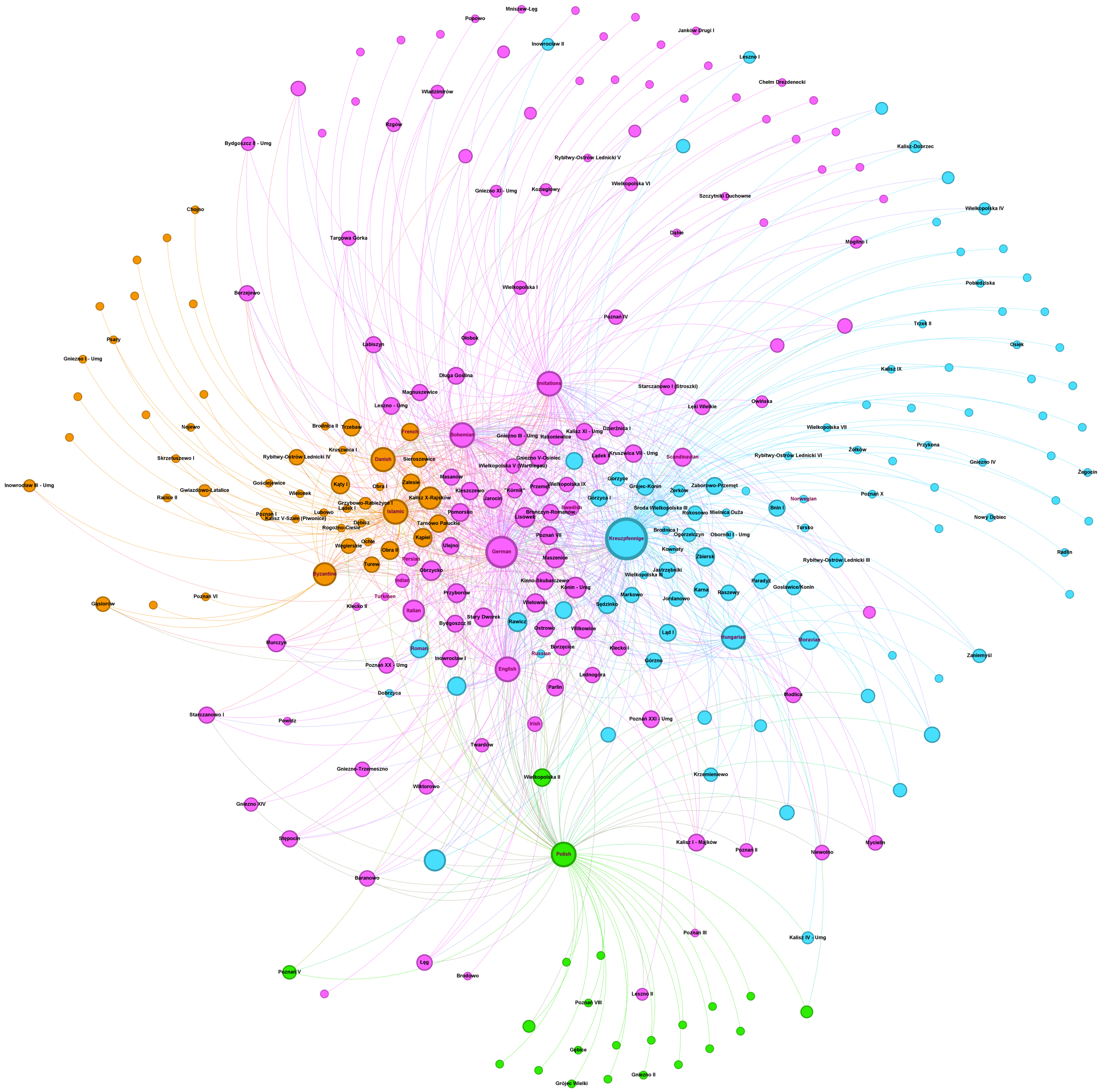

Fig. S4. The graph visualization of the social network analysis of silver hoards found in Greater Poland. Graph 2: The color of a node represents the community to which it has been assigned through modularity clustering method (Group 1 – orange, Group 2 – magenta, Group 3 – blue, Group 4 – green).

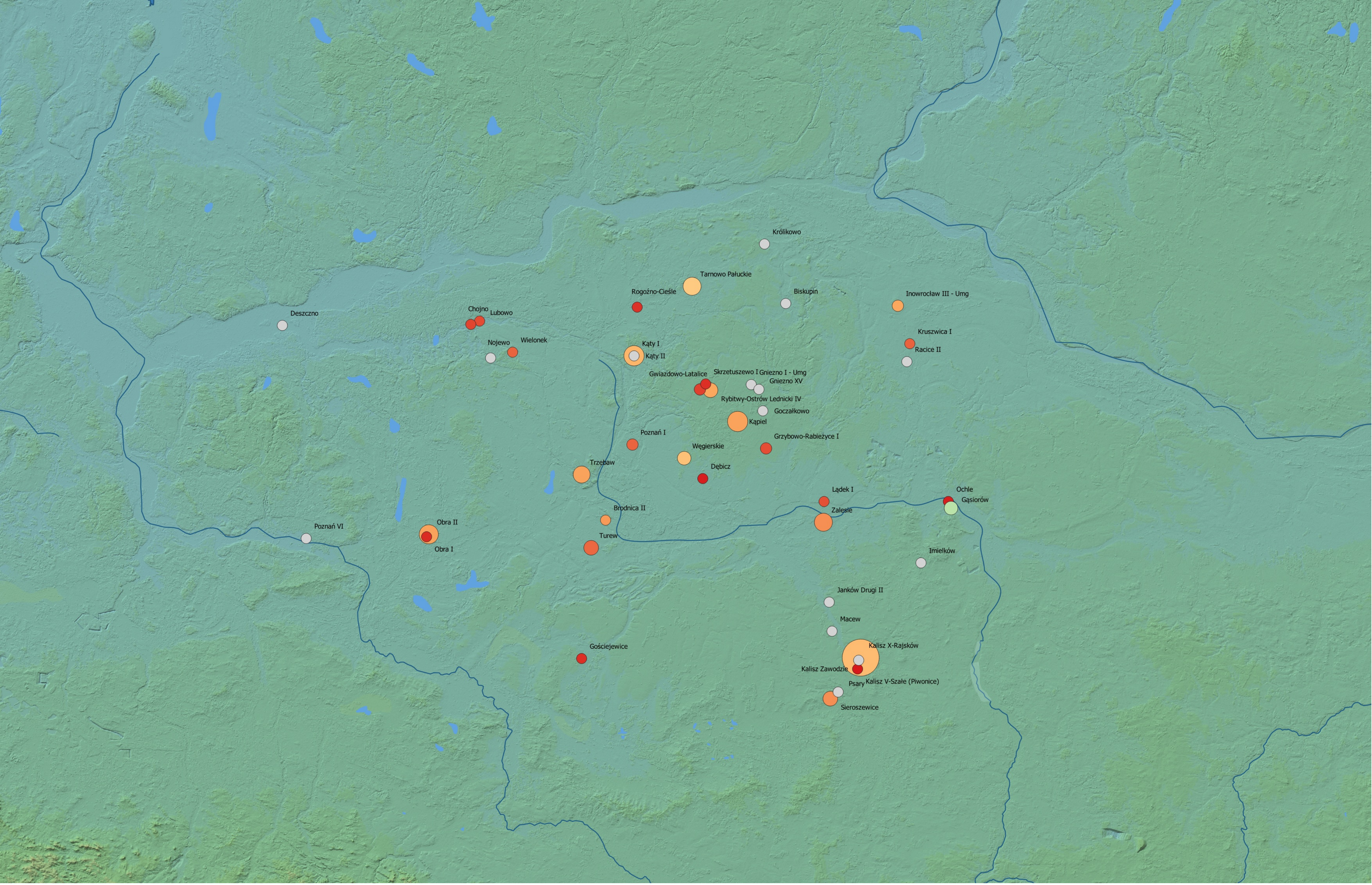

Hoard localizations: group 1

Fig. S5. The location of hoards assigned through modularity clustering to Group 1. The colours of the circles represent hoards' *terminus post quem* and is the same as in Figure S3

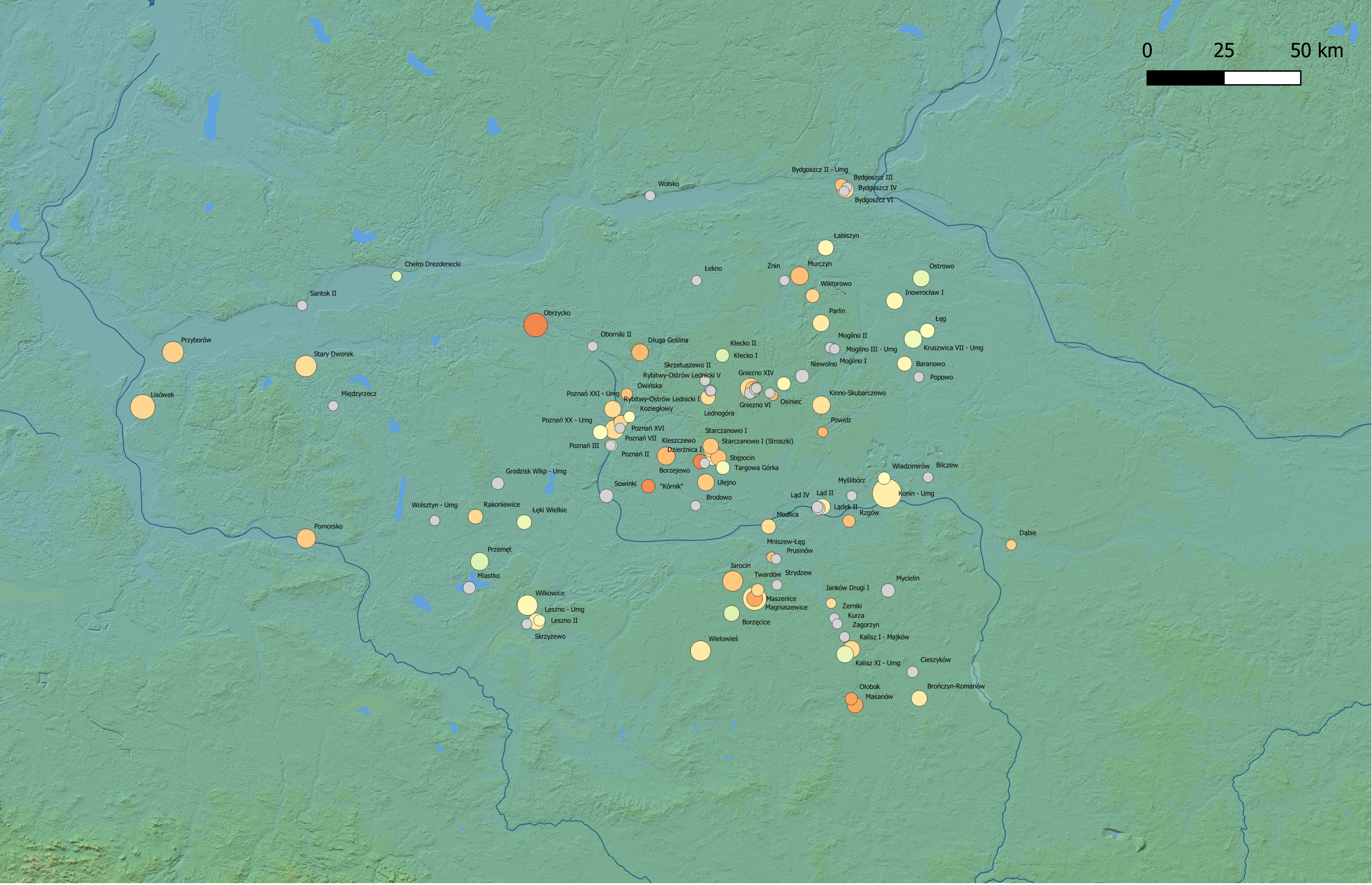

Hoard localizations: group 2

Fig. S6. The location of hoards assigned through modularity clustering to Group 2. The colours of the circles represent hoards' *terminus post quem* and is the same as in Figure S3

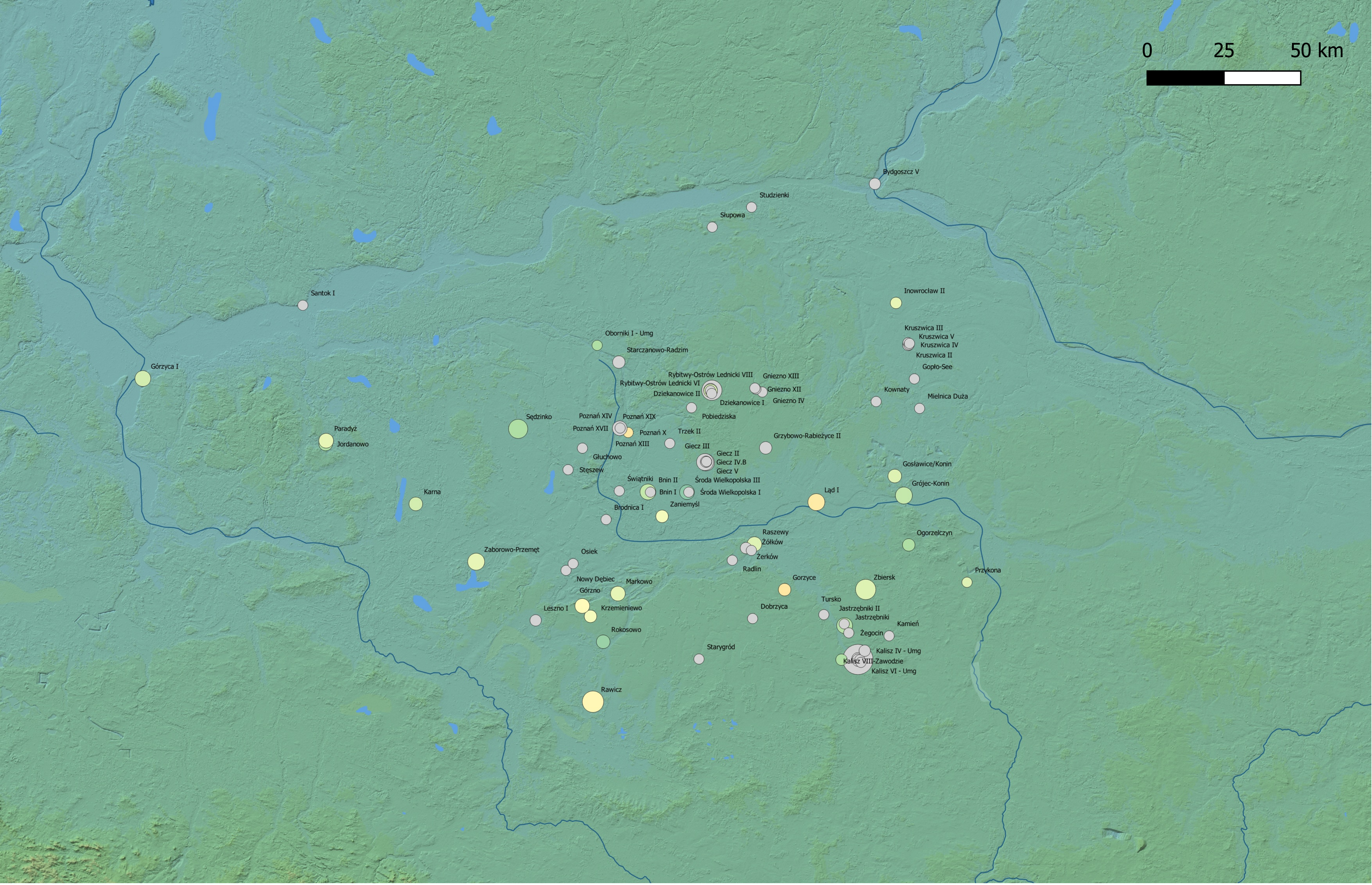

Hoard localizations: Group 3

Fig. S7. The location of hoards assigned through modularity clustering to Group 3. The colours of the circles represent hoards' *terminus post quem* and is the same as in Figure S3

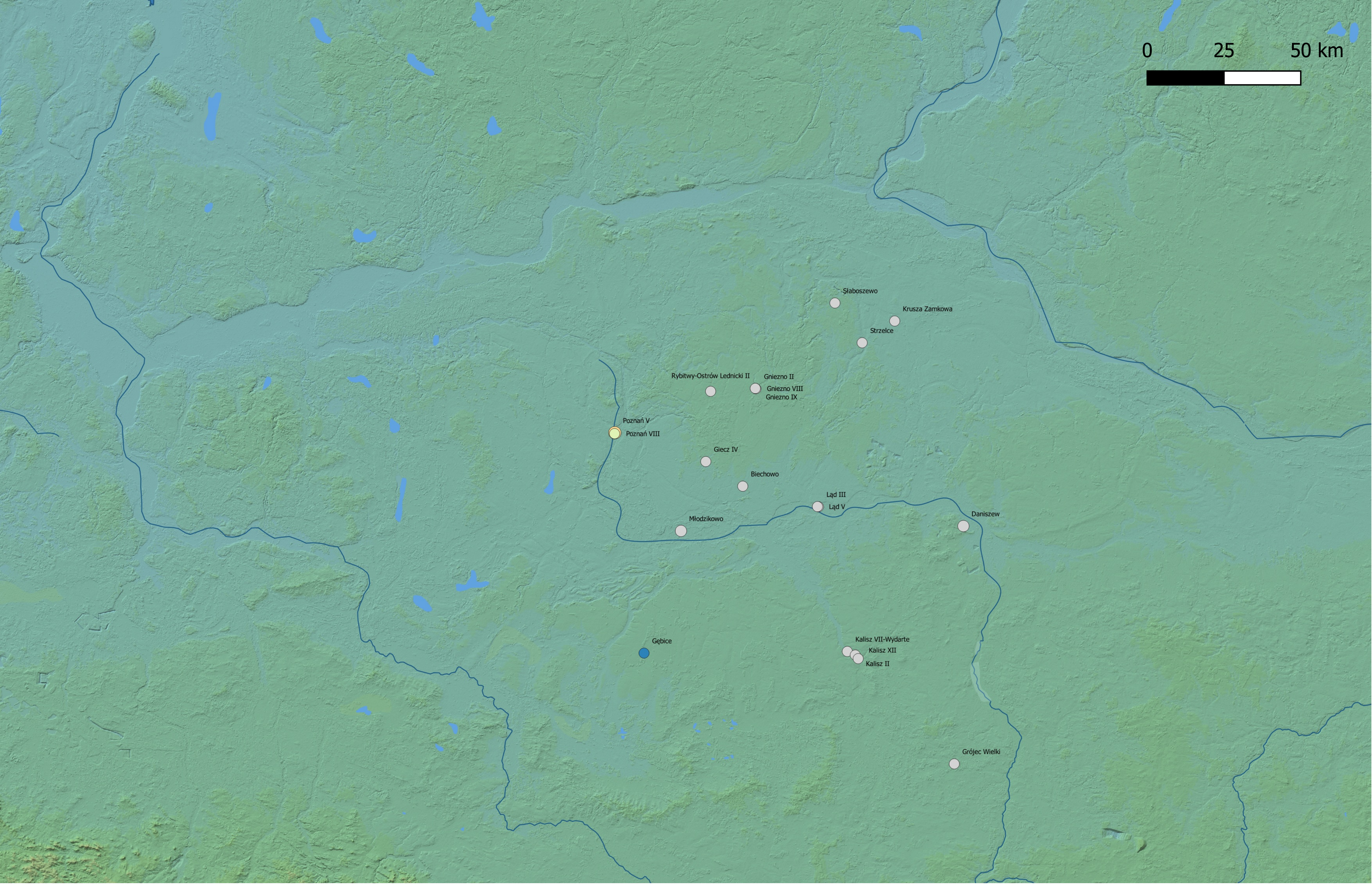

Hoard localizations: group 4

Fig. S8. The location of hoards assigned through modularity clustering to Group 4. The colours of the circles represent hoards' *terminus post quem* and is the same as in Figure S3

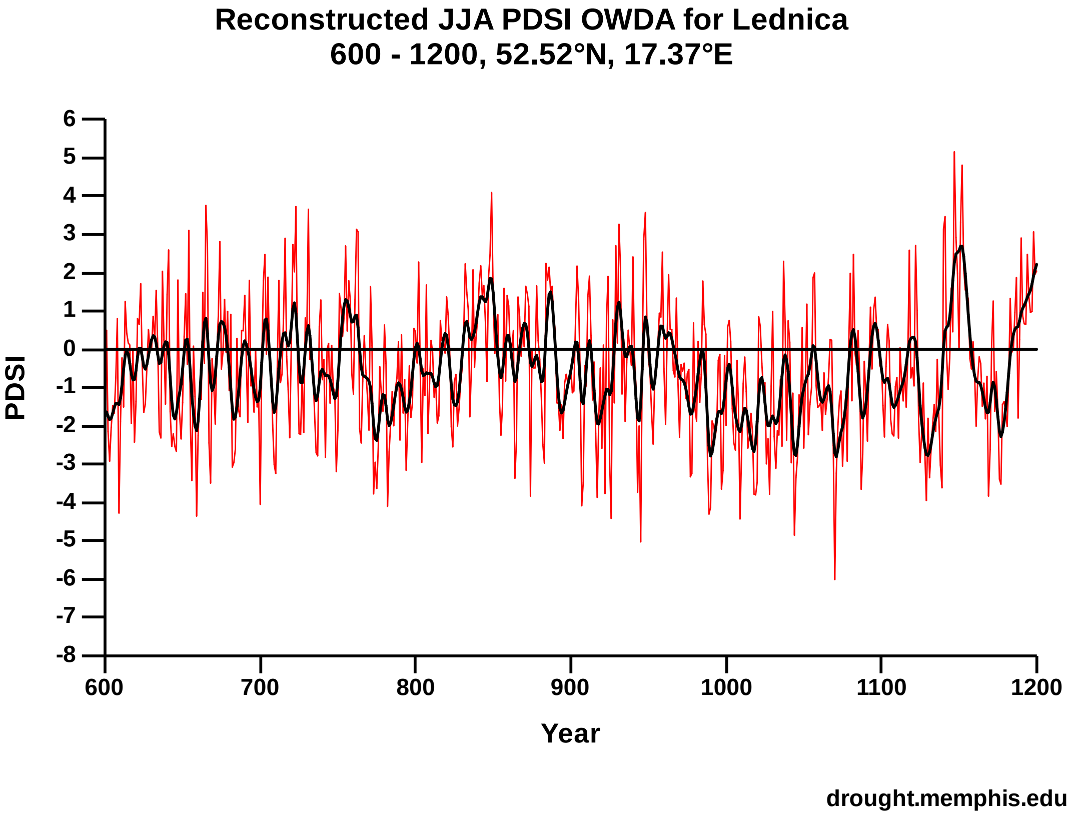

Fig. S9. Reconstructed 600-1200 CE June-July-August hydroclimate (PDSI) for Lednica, based on the Old World Drought Atlas (http://drought.memphis.edu). Data: Cook, E.R., et al., 2015: Old World megadroughts and pluvials during the Common Era. *Science Advances*, **1**, doi: 10.1126/sciadv.1500561.

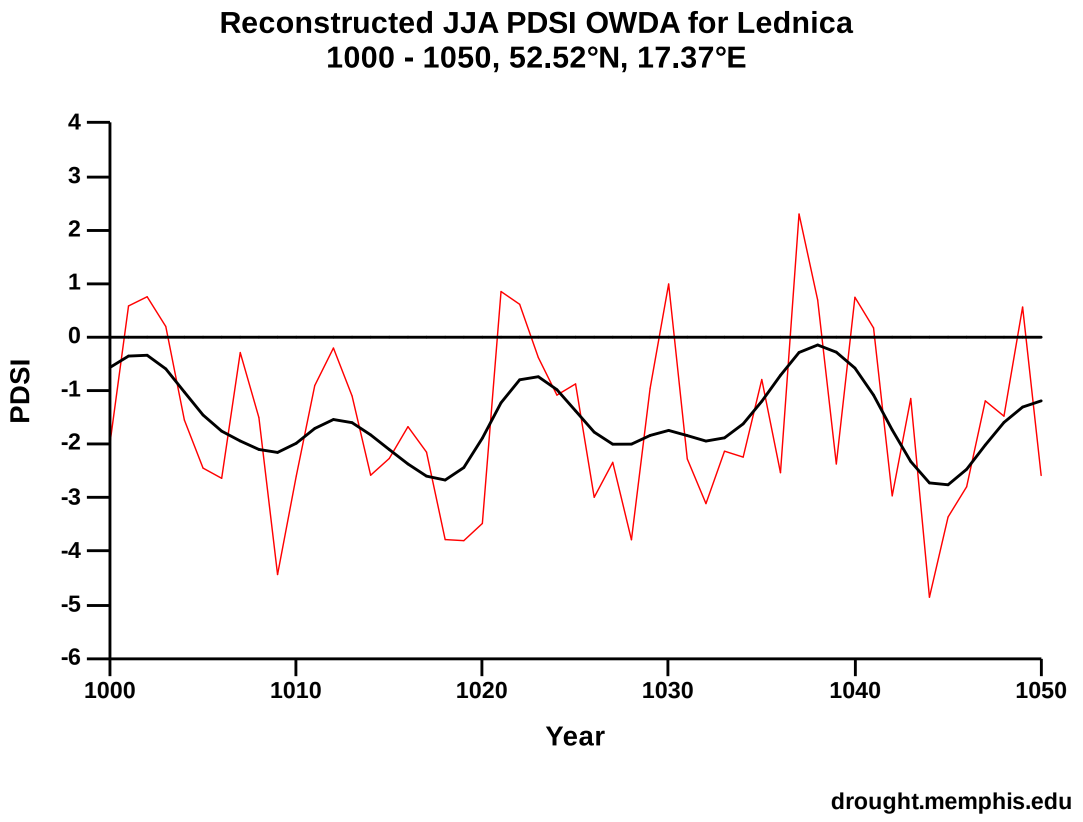

Fig. S10. Reconstructed 1000-1050 CE June-July-August hydroclimate (PDSI) for Lednica, based on the Old World Drought Atlas (http://drought.memphis.edu). Data: Cook, E.R., et al., 2015: Old World megadroughts and pluvials during the Common Era. *Science Advances*, **1**, doi: 10.1126/sciadv.1500561.

Table S1. ^{14}C dates from the Lednica core. Red color indicates dates excluded from the model.

N	Code	14C [BP]	Error	Depth [cm]	Dated material
1	ETH-121213	285	59	1.5	Plant epiderms
2	ETH-121211	234	66	9.5	Plant epiderms
3	ETH-121210	125	72	15.5	Plant epiderms
4	ETH-127528	344,8	56,9	19.5	Plant epiderms
5	ETH-121209	429	61	23.5	Plant epiderms
6	ETH-121205	188	93	31.5	Plant epiderms
7	ETH-121202	211	84	39.5	Plant epiderms
8	ETH-127529	404,8	52,9	49.5	Plant epiderms
9	ETH-121199	733	73	53.5	Plant epiderms
10	ETH-127530	1321,3	53,3	57.5	Plant epiderms
11	ETH-121197	1592	66	59.5	Plant epiderms
12	ETH-121193	733	71	65.5	Plant epiderms
13	ETH-121192	448	62	71.5	Plant epiderms
14	ETH-127531	785,1	52,3	73.5	Plant epiderms
15	ETH-127532	662,7	55,4	81.5	Plant epiderms
16	ETH-121189	371	81	85.5	Plant epiderms
17	ETH-127533	921,4	62,4	87.5	Plant epiderms
18	ETH-121212	600	69	95.5	Plant epiderms
19	ETH-121208	885	87	104.5	Plant epiderms
20	ETH-121207	1194	62	108.5	Plant epiderms
21	ETH-121206	1150	66	114.5	Plant epiderms
22	ETH-121204	1267	66	120.5	Plant epiderms
23	ETH-121203	1278	76	128.5	Plant epiderms
24	ETH-127534	1336,6	55,2	134.5	Plant epiderms
25	ETH-121201	1368	92	136.5	Plant epiderms
26	ETH-121198	1212	79	146.5	Plant epiderms
27	ETH-121196	1141	146	154.5	Plant epiderms
28	ETH-127535	854,5	53,3	158.5	Plant epiderms
29	ETH-121195	960	159	162.5	Plant epiderms
30	ETH-127536	966,7	55,1	164.5	Plant epiderms
31	ETH-127537	1233,7	53,1	174.5	Plant epiderms
32	ETH-121191	1447	69	176.5	Plant epiderms
33	ETH-127538	1063,8	63,5	186.5	Plant epiderms
34	ETH-127539	1458,2	59,8	196.5	Plant epiderms
35	Poz-167901	1780	120	221.5	Plant epiderms
36	Poz-167902	1955	35	234.5	Plant epiderms
37	Poz-167900	1970	70	249.5	Plant epiderms

Dataset S1 (separate file). Hoards dataset for Greater Poland, 9th-11th c. CE. Based on (43).

Dataset S2 (separate file). Strongholds dataset for Greater Poland, 9th-11th c. CE, based on several sources (see the file for more information).

SI References

(1) M. Makohonienko, “Materiały do postglacjalnej historii roślinności okolic Lednicy. Część II: Badania palinologiczne osadów Jeziora Lednickiego – rdzeń I/86 i WaI/87” in Wstęp do paleoekologii Lednickiego Parku Krajobrazowego, K. Tobolski, Ed. (Wydawnictwo Naukowe Uniwersytetu im. Adama Mickiewicza w Poznaniu, 1991), pp. 63-67.

(2) M. Makohonienko, *Przyrodnicza historia Gniezna* (Homini, 2000).

(3) T. Litt, K. Tobolski, “Materiały do postglacjalnej historii roślinności okolic Lednicy. Część II: Badania palinologiczne osadów Jeziora Lednickiego – rdzeń V/86“ in Wstęp do paleoekologii Lednickiego Parku Krajobrazowego, K. Tobolski, Ed. (Wydawnictwo Naukowe Uniwersytetu im. Adama Mickiewicza w Poznaniu, 1991), pp. 57-62.

(4) Tobolski, K. Paläoökologische Untersuchungen des Siedlungsgebietes im Lednica Landschaftspark (Nordwestpolen). Offa 47, 109–131 (1990).

(5) K. Tobolski, “Dotychczasowy stan badań paleobotanicznych i biostratygraficznych Lednickiego Parku Krajobrazowego“ in Wstęp do paleoekologii Lednickiego Parku Krajobrazowego, K. Tobolski, Ed. (Wydawnictwo Naukowe Uniwersytetu im. Adama Mickiewicza w Poznaniu, 1991), pp. 11-34.

(6) M.G.L. Baille, Suck-in and smear: two related chronological problems for the 90s. *J Theoretical Archaeology* 2, 12-16 (1991).

(7) M. Blaauw, J.A. Christen, D. Mauquoy, J. van der Plicht, K.D. Bennett, Testing the timing of radiocarbon-dated events between proxy archives. *The Holocene* 17, 283-288 (2007).

(8) L. Dumayne, R. Stoneman, K. Barber, D. Harkness, Problems associated with correlating calibrated radiocarbon-dated pollen diagrams with historical events. *The Holocene* 5, 118-123 (1995).

(9) S. Czerwiński, K. Marcisz, A. Wacnik, M. Lamentowicz, Synthesis of palaeoecological data from the Polish Lowlands suggests heterogeneous patterns of old-growth forest loss after the Migration Period. *Scientific Reports* 12, 8559 (2022).

(10) M. Ralska-Jasiewiczowa, D. Nalepka, T. Goslar, Some problems of forest transformation at the transition to the oligocratic/Homo sapiens phase of the Holocene interglacial in northern lowlands of central Europe. *Vegetation History and Archaeobotany* 12, 233-247 (2003).

(11) A. Filbrandt, “Badania paleoekologiczne osadów limnicznych jeziora Kamionek” in Wstęp do paleoekologii Lednickiego Parku Krajobrazowego, K. Tobolski, Ed. (Wydawnictwo Naukowe Uniwersytetu im. Adama Mickiewicza w Poznaniu, 1991), pp. 81-86.

(12) K. Milecka, *Historia działalności człowieka w okolicach Giecza i Wagowa w świetle analizy pyłkowej* (Wydawnictwo Naukowe Uniwersytetu im. Adama Mickiewicza w Poznaniu, 1998).

(13) K. Milecka, “Analiza pyłkowa osadów jeziora w Gieczu” in Wstęp do paleoekologii Lednickiego Parku Krajobrazowego, K. Tobolski, Ed. (Wydawnictwo Naukowe Uniwersytetu im. Adama Mickiewicza w Poznaniu, 1991), pp. 147-150.

(14) M. Moździoch, “From a tribe to a state” in The Past Societies. Polish lands from the first evidence of human presence to the Early Middle Ages Vol. 5: 500–1000 AD, P. Urbańczyk, M. Trzeciecki, Eds. (The Institute of Archaeology and Ethnology, Polish Academy of Sciences, 2016), pp. 123-167.

(15) M. Wołoszyn, “The Migration Period in Poland in the Light of Literary Sources” in The Migration Period between the Oder and the Vistula, A. Bursche, J. Hines, A. Zapolska (Brill, 2020), pp. 84-136.

(16) A. Pędziszewska, M. Latałowa, J. Święta-Musznicka, M. Zimny, M. Kupryjanowicz, A.M. Noryśkiewicz, K. Bloom, “Pollen Evidence of Change in Environment and Settlement during the 1st Millennium AD” in The Migration Period between the Oder and the Vistula, A. Bursche, J. Hines, A. Zapolska (Brill, 2020), pp. 137-198.

(17) P. Beck, G. Caudullo, W. Tinner, D. de Rigo, “Fraxinus excelsior in Europe: distribution, habitat, usage and threats” in European Atlas of Forest Tree Species, J. San-Miguel-Ayanz et al., Eds. (Publication Office of the EU, 2016), pp. e0181c0+.

(18) K. Cywa, Trees and shrubs used in medieval Poland for making everyday objects. *Vegetation History and Archaeobotany* 27, 111-136 (2018).

(19) S. Czerwiński, P. Guzowski, M. Lamentowicz, M. Gałka, M. Karpińska-Kołaczek, R. Poniat, E. Łokas, A.-C. Diaconu, J. Schwarzer, M. Miecznik, P. Kołaczek, Environmental implications of past socioeconomic events in Greater Poland during the last 1200 years. Synthesis of paleoecological and historical data. *Quaternary Science Reviews* 259 (2021).

(20) C. Cugny, F. Mazier, D. Galop, Modern and fossil non-pollen palynomorphs from the Basque mountains (western Pyrenees, France): the use of coprophilous fungi to reconstruct pastoral activity. *Vegetation History and Archaeobotany* 19, 391-408 (2010).

(21) M. Krąpiec, Oak dendrochronology of the neoholocene in Poland. *Folia Quat.*, 69, 5-133 (1998).

(22) M. Trzeciecki, “The emergence of the territorial state” in The Past Societies. Polish lands from the first evidence of human presence to the Early Middle Ages Vol. 5: 500–1000 AD, P. Urbańczyk, M. Trzeciecki, Eds. (The Institute of Archaeology and Ethnology, Polish Academy of Sciences, 2016), pp. 277-341.

(23) M. Hillbrand, B. van Geel, A. Hasenfratz, P. Hadorn, J.N. Haas, Non-pollen palynomorphs show human- and livestock-induced eutrophication of Lake Nussbaumersee (Thurgau, Switzerland) since Neolithic times (3840 bc). *The Holocene*, 24(5), 559-568 (2014).

(24) T. Związek, *et al.*, On the economic impact of droughts in central Europe: the decade from 1531 to 1540 from the Polish perspective. *Climate of the Past* 18, 1541–1561 (2022).

(25) K. Wnęk, A. Izdebski, L. Kowanetz, “The climate history of Krakow” in *Krakow. An Ecobiography*, History of the urban environment., A. Izdebski, R. Szmytka, Eds. (Pittsburgh University Press, 2022), pp. 22–42.

(26) Cook, E.R., et al., 2015: Old World megadroughts and pluvials during the Common Era. *Science Advances*, 1, doi: 10.1126/sciadv.1500561

(27) M. Gałka, *et al.*, Palaeohydrology, fires and vegetation succession in the southern Baltic during the last 7500 years reconstructed from a raised bog based on multi-proxy data. *Palaeogeography, Palaeoclimatology, Palaeoecology* 370, 209 – 221 (2013).

(28) M. Lamentowicz, *et al.*, How Joannites’ economy eradicated primeval forest and created anthroecosystems in medieval Central Europe. *Sci Rep* 10 (1), 18775 (2020).

(29) Marcisz K., *et al.*, Long-term hydrological dynamics and fire history over the last 2000 years in CE Europe reconstructed from a high-resolution peat archive. *Quaternary Science Reviews* 112, 138–152 (2015).

(30) U. Büntgen, *et al.*, 2500 Years of European Climate Variability and Human Susceptibility. *Science* 331, 578–582 (2011).

(31) J. Luterbacher, *et al.*, European summer temperatures since Roman times. *Environ. Res. Lett.* 11, 024001 (2016).

(32) U. Büntgen, *et al.*, Filling the Eastern European gap in millennium-long temperature reconstructions. *PNAS* 110, 1773–1778 (2013).

(33) M. Toohey, M. Sigl, Volcanic stratospheric sulfur injections and aerosol optical depth from 500 BCE to 1900 CE. *Earth System Science Data* 9, 809–831 (2017).

(34) C. Oppenheimer, *et al.*, The Eldgjá eruption: timing, long-range impacts and influence on the Christianisation of Iceland. *Climatic Change* 147, 369–381 (2018).

(35) J. Topolski, “Wzrost gospodarczy i jego bariery. Aktywizacja ekonomiczna szlachty”, in *Dzieje Wielkopolski*, J. Topolski, Ed. (Wydawnictwo Poznańskie 1969), p. 444.

(36) H. Łowmiański, *Początki Polski. Z dziejów Słowian w I tysiącleciu* (Państwowe Wydawnictwo Naukowe 1967), vol. IV, p. 296.

(37) A. Wyczański, *Studia nad konsumpcją żywności w Polsce w XVI i pierwszej połowie XVII w.* (Państwowe Wydawnictwo Naukowe, 1969), s. 186.

(38) T. Ładogórski, Zaludnienie ziem polskich w czasach Bolesława Chrobrego, *Roczniki Dziejów Społecznych i Gospodarczych* 50, 21-29 (1980).
(39) P. Lunde, C. Stone, *Ibn Fadlān and the Land of Darkness: Arab Travellers in the Far North* (Penguin, 2012).
(40) C. B. Ramsey, Bayesian Analysis of Radiocarbon Dates. *Radiocarbon* 51(1), 337-360 (2009).
(41) P. J. Reimer *et al.*, The IntCal20 Northern Hemisphere Radiocarbon Age Calibration Curve (0–55 cal kBP), *Radiocarbon* 62(4), 725-757 (2020).
(42) E.C. Grimm, CONISS: a FORTRAN 77 program for stratigraphically constrained cluster analysis by the method of incremental sum of squares, *Comput. Geosci.* 13, 13-35 (1987).
(43) M. Bogucki, P. Ilisch and S. Suchodolski, *Frühmittelalterliche Münzfunde aus Polen : Inventar*, vol. 1: *Grosspolen* (Institut für Archäologie und Ethnologie der Polnischen Akademie der Wissenschaften, 2017).
